# Giant chiral amplification of chiral 2D perovskites via dynamic crystal reconstruction

**DOI:** 10.1126/sciadv.ado5942

**Published:** 2024-08-21

**Authors:** Hongki Kim, Wonbin Choi, Yu Jin Kim, Jihoon Kim, Jaeyong Ahn, Inho Song, Minjoon Kwak, Jongchan Kim, Jonghyun Park, Dongwon Yoo, Jungwon Park, Sang Kyu Kwak, Joon Hak Oh

**Affiliations:** ^1^ School of Chemical and Biological Engineering, Institute of Chemical Processes, Seoul National University, Seoul 08826, Republic of Korea.; ^2^ Department of Applied Physical Sciences, University of North Carolina at Chapel Hill, Chapel Hill, NC, USA.; ^3^ School of Energy and Chemical Engineering, Ulsan National Institute of Science and Technology (UNIST), Ulsan 44919, Republic of Korea.; ^4^ Center for Nanoparticle Research, Institute for Basic Science (IBS), Seoul National University, Seoul 08826, Republic of Korea.; ^5^ Department of Chemistry, Purdue University, West Lafayette, IN, USA.; ^6^ Department of Chemical and Biological Engineering, Korea University, Seoul 02841, Republic of Korea.

## Abstract

Chiral hybrid perovskites show promise for advanced spin-resolved optoelectronics due to their excellent polarization-sensitive properties. However, chiral perovskites developed to date rely solely on the interaction between chiral organic ligand cations exhibiting point chirality and an inorganic framework, leading to a poorly ordered short-range chiral system. Here, we report a powerful method to overcome this limitation using dynamic long-range organization of chiral perovskites guided by the incorporation of chiral dopants, which induces strong interactions between chiral dopants and chiral cations. The additional interplay of chiral cations with chiral dopants reorganizes the morphological and crystallographic properties of chiral perovskites, notably enhancing the asymmetric behavior of chiral 2D perovskites by more than 10-fold, along with the highest dissymmetry factor of photocurrent (*g*_Ph_) of ~1.16 reported to date. Our findings present a pioneering approach to efficiently amplify the chiroptical response in chiral perovskites, opening avenues for exploring their potential in cutting-edge optoelectronic applications.

## Teaser

Triple interplay between inorganic sublattices, chiral organic cations, and chiral dopants enables giant chiral amplification.

## INTRODUCTION

Chiral materials interact strongly with the spin angular momentum (SAM) of photons when subjected to chiral electromagnetic fields, giving rise to circular dichroism (CD) or circularly polarized photoluminescence (CPPL). The former is the anisotropic absorption between right-handed circularly polarized light (RCPL) and left-handed circularly polarized light (LCPL), and the latter is due to anisotropic emission between RCPL and LCPL (*1*, *2*). Integrating these functionalities into optoelectronic devices allows for various next-generation applications, ranging from chiral recognition and polarized imaging to optical quantum cryptography and optical communication (*3*–*8*). Therefore, there is a great deal of interest in constructing efficient SAM-active layers and leveraging them in cutting-edge applications.

Chiral two-dimensional (2D) organic-inorganic hybrid perovskites (OIHPs) are emerging as a promising class of SAM-active materials for chiral optoelectronics (e.g., CPL detectors or CPL emitters) due to their efficient spin-polarized charge transport, high absorption coefficient, and tunable bandgap (*9*, *10*). Nevertheless, their weak optical activity and limited methodologies for boosting their chiroptical activity remain major obstacles that must be addressed to fully explore their potential applications. Chiroptical properties in existing chiral 2D OIHPs have primarily been driven by chiral transfer from chiral organic cations to inorganic sublattices (*2*). However, the coherent orientation of chiral organic cations is hindered by weak π-π interactions in typical chiral 2D OIHPs, leading to inhomogeneous chirality transfer from chiral organic ligand cations to inorganic frameworks. Moreover, the lack of control over their ordering within inorganic frameworks during fast crystallization processes leads to the inevitable production of a poorly ordered chiral 2D OIHP system. This indicates an intrinsic limitation of conventional monolithic chiral organic cation–inorganic framework systems (MCIFs), driven solely by the monotonous interplay between chiral organic cations and inorganic frameworks.

Several approaches to enhance and control the chiroptical properties of chiral 2D OIHPs have been developed: tailoring the molecular structure of chiral organic cations using a variety of chiral cations, such as *R* or *S*-methylbenzylammonium (*R* or *S*-MBA^+^) (*11*–*16*), *R* or *S*-methylphenethylammonium (*R* or *S*-MPA^+^) (*17*, *18*), *R* or *S*-(naphthyl)ethylammonium (*R* or *S*-NEA^+^) (*19*, *20*), and halide substituted *R* or *S*-*X-*MBA^+^ (*X* = F, Cl, Br, I) (*21*–*23*); utilization of blends of chiral organic cation and achiral organic cations (*24*–*26*); introduction of subsidiary nanoporous templates or cavities (*27*–*29*); dimensionality engineering of perovskites (*19*, *30*); introduction of additives (*31*); and controlling the compositional elements of perovskites (*32*, *33*). However, these approaches have drawbacks, such as undesired inversion or peak shifts in the original CD spectra, challenges in device integration due to the introduction of extra structures (i.e., templates or cavities), and inefficient chiral amplification. Therefore, a powerful methodology is required to enhance the chirality of 2D perovskites in a more efficient and controllable manner while preserving the distinctive characteristics (such as sign and peak position) evident in the CD spectra.

Beyond the conventional MCIFs, we herein introduce additional chiral dopants [(2*S*,3*S*/2*R*,3*R*)-2,3-*O*-isopropylidene-1,1,4,4-tetraphenyl-1,2,3,4-butanetetrol, i.e., (+/−)-TADDOLs] into chiral 2D OIHPs with chiral organic ligand cations (*R*/*S*-MBA^+^). The additional interplay between chiral organic cations and chiral dopants leads to the dynamic crystal reconstruction in chiral 2D OIHPs, giving rise to intriguing phenomena. First, the appearance of unique spherulitic tornado-like morphologies with high homogeneity enables a long-range chiral activity. Second, crystals in chiral 2D OIHPs undergo splintering into much smaller crystals under a compressive strain, promoting the lattice distortion in the inorganic frameworks. As a consequence of these advancements, the anisotropy factor of chiral 2D OIHP films with TADDOLs exhibits a remarkable chiral amplification effect without signal inversion or substantial shift on the CD spectra. Last, we have successfully implemented a self-powered CPL detector, achieving an outstanding *g*_Ph_ surpassing 1. These results demonstrate a highly efficient and viable pathway to overcome the limitations of typical MCIFs by introducing a chiral dopant.

## RESULTS

### Long-range organization of chiral 2D OIHPs guided by chiral dopants

**Fig. 1.**
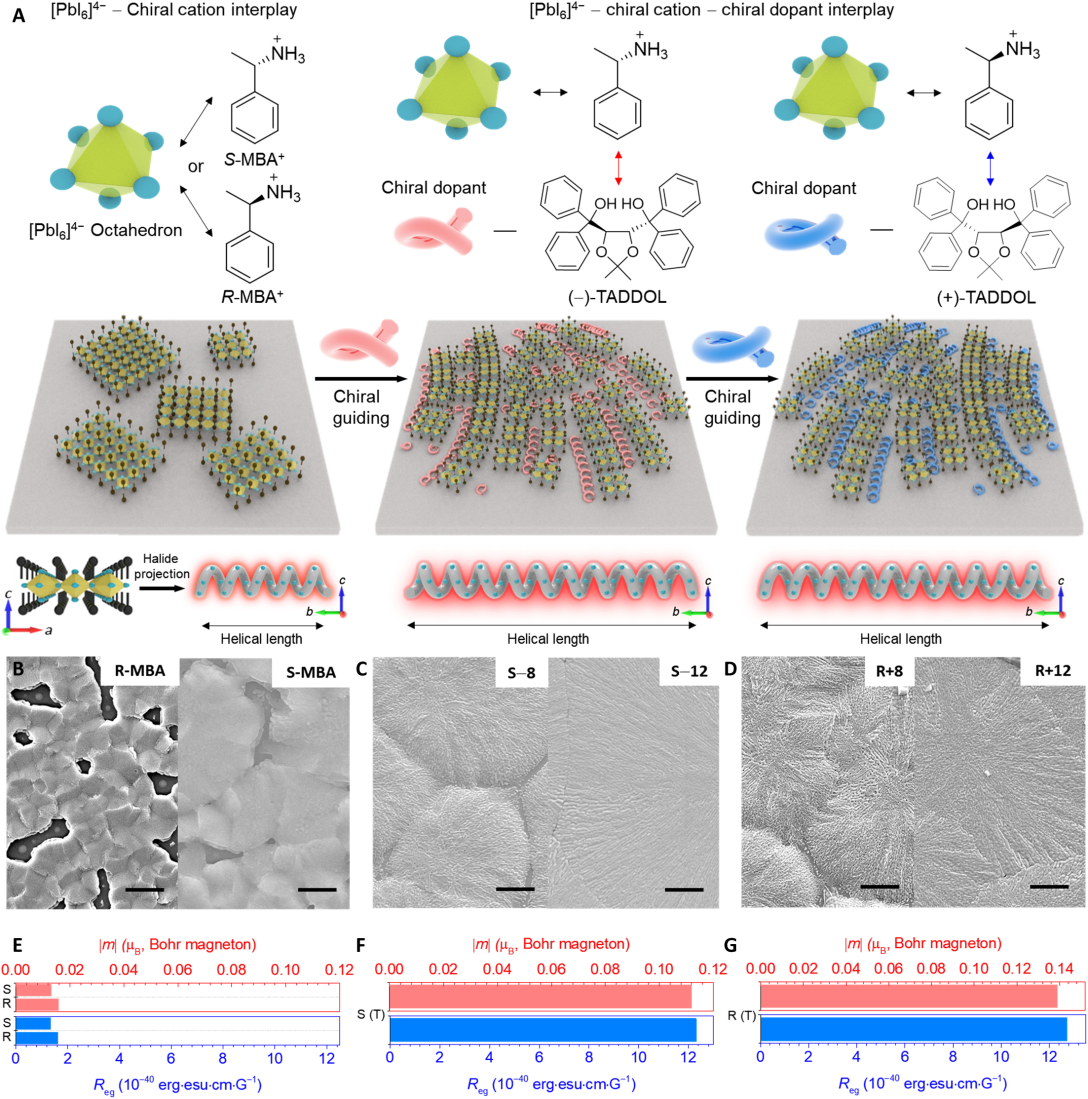
Long-range chiral model of chiral 2D OIHPs with TADDOLs. (**A**) Schematic illustration of typical chiral 2D OIHPs driven by mono-interplay between chiral organic cations and inorganic frameworks and their evolution to long-range chiral assemblies driven by the additional interplay between chiral organic cations and TADDOLs. (**B** to **D**) SEM images of (B) pristine (*S*/*R*-MBA)_2_PbI_4_ films, (C) (−)-TADDOL (8 or 12%)–introduced (*S*-MBA)_2_PbI_4_ and (D) (+)-TADDOL (8 or 12%)–introduced (*R*-MBA)_2_PbI_4_ films. Inset scale bars in SEM images represent 2 μm. (**E** to **G**) Estimated |*m*| and *R*_eg_ of (E) pristine (*S*/*R*-MBA)_2_PbI_4_ films and (F) TADDOL-introduced (*S*-MBA)_2_PbI_4_ and (G) (*R*-MBA)_2_PbI_4_ films.

Unlike typical chiral cations (*R*/*S*-MBA^+^) with point chirality, a secondary chiral component (TADDOL) enables self-assembly to produce long-range chirality and distinctive morphologies due to the intermolecular interactions encompassing hydrogen bonding and π-π stacking (figs. S1 and S2). Harnessing the unique properties of TADDOLs, we used a systematic design approach, hypothesizing that the morphological organization of chiral 2D OIHPs could potentially differ after introducing the TADDOLs compared to MCIFs. This divergence may occur given the potential additional interplay between TADDOLs and chiral organic cations (Fig. 1A).

To confirm this hypothesis, we investigated the morphologies of TADDOL-introduced (*R*/*S*-MBA)_2_PbI_4_ films via scanning electron microscopy (SEM). After material verification on the single-crystal chiral 2D OIHP (fig. S3), we added TADDOLs into (*R*/*S*-MBA)_2_PbI_4_ with controlled TADDOL-to-*R*/*S*-MBA^+^ molar ratios. Upon adding (+)-TADDOL or (−)-TADDOL into (*R*-MBA)_2_PbI_4_ or (*S*-MBA)_2_PbI_4_, spherulitic tornado-like morphologies appeared, in stark contrast to the pristine films (Fig. 1, B to D, and fig. S4). Without their introduction, (*R*/*S*-MBA)_2_PbI_4_ thin films consisted of grains with discernible grain size and general shape. This morphology produced many pinholes in the film. On the other hand, chiral 2D OIHP films showed entirely reconstructed morphological properties after their introduction. Crystals were splintered into much smaller crystals with sizes that were difficult to discern, and a spherulitic tornado-like morphology extending from the central point was produced with much greater compactness and higher homogeneity compared to the pristine film. When adding (−)-TADDOL or (+)-TADDOL into (*R*-MBA)_2_PbI_4_ or (*S*-MBA)_2_PbI_4_, respectively, similar morphologies were observed with splintered grains but with a slightly blurred spherulitic tornado-like morphology (fig. S5). The smaller crystal size with the addition of TADDOL was also confirmed via a transmission electron microscope (TEM) (fig. S6).

We attributed this to the effective suppression of macrocrystal formation through the interaction between TADDOLs and chiral amines, which facilitates the reconstruction of splintered crystals in a similar manner to the self-assembly of TADDOLs. Because of the presence of a halide helical screw parallel to the substrate in the symmetry-breaking distorted inorganic framework (fig. S7) (*34*), the introduction of TADDOLs and their homogeneous coassembly with chiral 2D OIHPs enables long-range chiral order with extended helical length of the halide screw. This eventually increases the local magnitude of magnetic fields (*B*), directly contributing to the anisotropy factor (*35*, *36*). To scrutinize this phenomenon, we calculated the magnetic transition dipole moment (|*m*|) and the rotational strength (*R*_eg_) by obtaining the electric transition dipole moment (μ) and the asymmetry factor of CPPL (*g*_PL_) (note S1, fig. S8, and table S1). Highly amplified |*m*| and *R_eg_* were obtained with the introduction of TADDOLs, intensifying |*m*| of (*R*-MBA)_2_PbI_4_ [(*S*-MBA)_2_PbI_4_] from 0.0158 μ_B_ (0.0131 μ_B_) to 0.1388 μ_B_ (0.1120 μ_B_) and *R_eg_* of (*R*-MBA)_2_PbI_4_ [(*S*-MBA)_2_PbI_4_] from 1.624 × 10^−40^ erg esu cm G^−1^ (1.333 × 10^−40^ erg esu cm G^−1^) to 12.731 × 10^−40^ erg esu cm G^−1^ (12.311 × 10^−40^ erg esu cm G^−1^) (Fig. 1, E to G).

### Chiroptical properties of chiral 2D OIHPs with chiral dopants

**Fig. 2.**
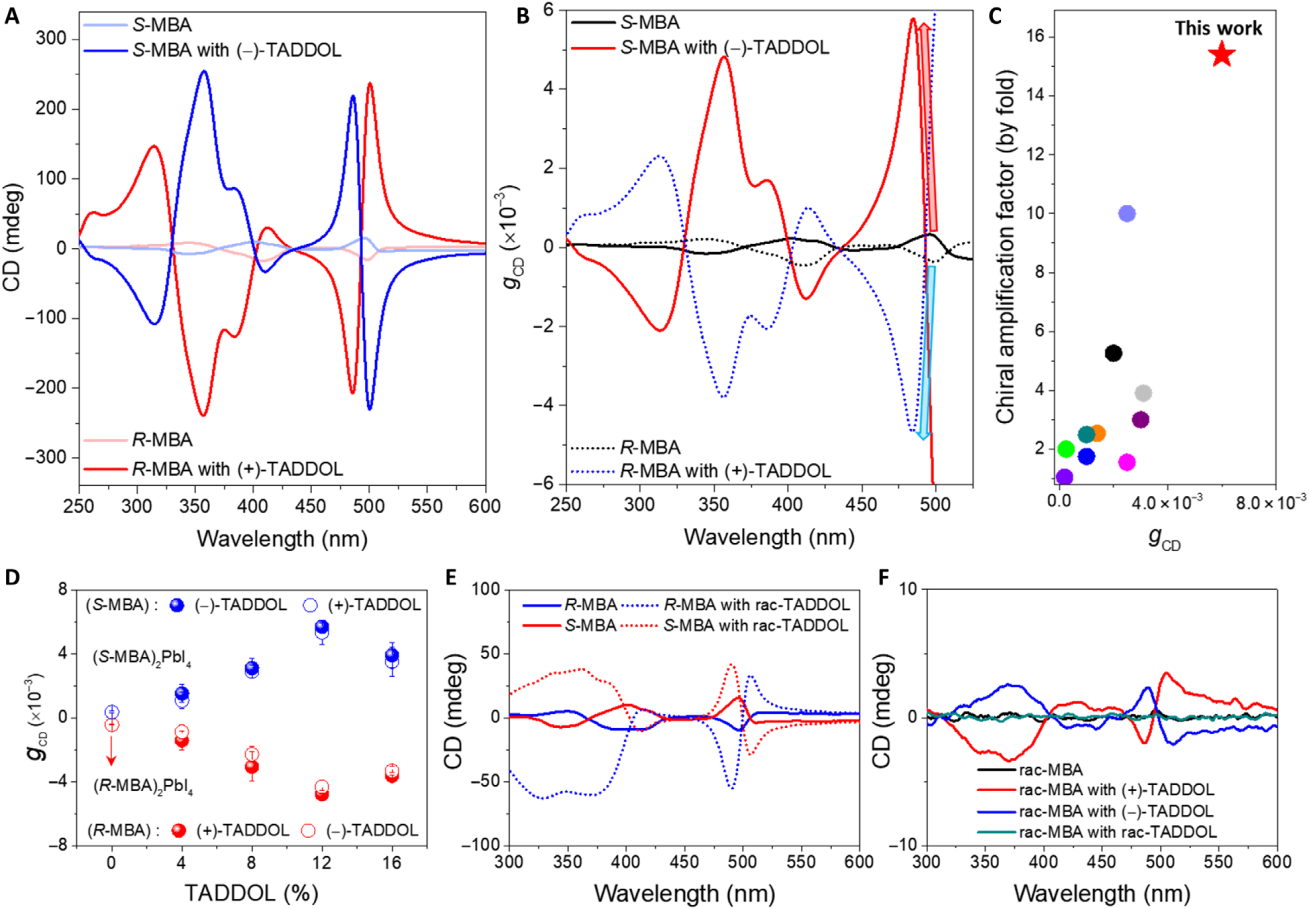
Chiroptical properties of chiral 2D OIHPs with TADDOLs. (**A** and **B**) (A) CD spectra of (*S*-MBA)_2_PbI_4_ and (*R*-MBA)_2_PbI_4_ with or without the introduction of TADDOLs and (B) their corresponding *g*_CD_ spectra. (**C**) Correlation between chiral amplification factors with *g*_CD_ in reported studies for chiral 2D OIHPs (see table S2). (**D**) Statistical data of *g*_CD_ with different concentrations and combinations of (+/−)-TADDOLs. (**E** and **F**) CD spectra of (E) chiral 2D OIHP thin films with racemic TADDOL and (F) racemic 2D OIHP thin film with (+/−)-TADDOLs.

Next, we studied the chiroptical properties of TADDOL-introduced (*R*/*S*-MBA)_2_PbI_4_ thin films. The features of the CD spectra of the reference (*R*/*S*-MBA)_2_PbI_4_ were consistent with previous results (fig. S9) (*21*, *32*), where the opposite signal values were obtained between (*R*-MBA)_2_PbI_4_ and (*S*-MBA)_2_PbI_4_. The CD intensities of (*R*/*S*-MBA)_2_PbI_4_ films were highly amplified after the introduction of TADDOLs (Fig. 2A). Specifically, the CD intensities of (−)-TADDOL–introduced (*S*-MBA)_2_PbI_4_ film and (+)-TADDOL–introduced (*R*-MBA)_2_PbI_4_ film were notably intensified from 15.576 mdeg at a wavelength of 495.5 nm to 219.117 mdeg at a wavelength of 486.0 nm and from −15.798 mdeg at a wavelength of 498 nm to −207.268 mdeg at a wavelength of 485.5 nm, respectively. The introduction of chiral molecules with the same structure as TADDOLs, except for the absence of benzene rings, did not amplify the chiral activity of chiral 2D OIHPs (fig. S10). These results suggest that chiral dopants with the π-π stacking of the benzene rings play a crucial role in amplifying chiral activity. We also confirmed that the chiral amplification did not originate from the optical interference of thin film’s linear birefringence (LB) and linear dichroism (LD) (i.e., LDLB effect), which can lead to the macroscopic anisotropic effect (*27*). To exclude LDLB effect, we observed the CD spectra of TADDOL-introduced chiral 2D OIHP films by flipping the sample by 180° with respect to the vertical axis. We confirmed that similar CD spectra were obtained when the sample was flipped (fig. S11), verifying the observed chiral amplification of TADDOL-introduced chiral 2D OIHP films originated from the intrinsic chiroptical property.

Chiroptical amplification was accompanied by several phenomena. First, weak hypsochromic shifts in absorbance were observed after introduction (fig. S12). This can be related to the induction of confinement in inorganic frameworks, which is discussed later. Second, exciton-coupled CD signals with bisignated Cotton effects strongly occurred. This was interpreted as a resonant coupling of excitons with the preferred magnitude and arrangement of the electric and magnetic transition dipole moments (*37*, *38*). Along with strong Cotton effects, the amplification of all CD peaks assigned to each exciton transition demonstrated the effectiveness of TADDOLs as chiral amplifiers (fig. S13). Highly amplified |*g*_CD_| of 4.68 × 10^−3^ [(*R*-MBA)_2_PbI_4_ with (+)-TADDOL] and 5.79 × 10^−3^ [(*S*-MBA)_2_PbI_4_ with (−)-TADDOL] were obtained compared to the |*g*_CD_| for both reference (*R*-MBA)_2_PbI_4_ (3.70 × 10^−4^) and (*S*-MBA)_2_PbI_4_ (3.30 × 10^−4^) (Fig. 2B), which increased by more than 10-fold. This enhancement was among the highest chiral amplification effects reported for chiral 2D perovskite films without the use of additional templates or cavities, and it was achieved without inversion or substantial changes in the CD spectra (Fig. 2C and table S2). Highly amplified chiral anisotropic behavior was also confirmed by amplified CPPL intensities, with an improvement in the |*g*_PL_| value from 0.53 × 10^−3^ (0.44 × 10^−3^) for (*R*-MBA)_2_PbI_4_ [(*S*-MBA)_2_PbI_4_] films to 0.46 × 10^−2^ (0.35 × 10^−2^) for (*R*-MBA)_2_PbI_4_ with (+)-TADDOL [(*S*-MBA)_2_PbI_4_ with (−)-TADDOL] films (fig. S14).

The *g*_CD_ values and CD spectra with various doping concentrations of (+/−)-TADDOL and different combinations between (+/−)-TADDOLs and (*R*/*S*-MBA)_2_PbI_4_ are summarized in Fig. 2D and fig. S15. A chirality amplification effect was clearly observed regardless of whether enantiomers of TADDOLs were used. (*R*-MBA)_2_PbI_4_ films exhibited slightly higher *g*_CD_ values when coupled with (+)-TADDOLs compared to (−)-TADDOLs. Conversely, (*S*-MBA)_2_PbI_4_ films showed slightly higher *g*_CD_ values when coupled with (−)-TADDOLs compared to (+)-TADDOLs. It is also noteworthy that CD intensities of (*R*/*S*-MBA)_2_PbI_4_ films were amplified after adding a racemic mixture of (+)-TADDOL and (−)-TADDOL (Fig. 2E), and the chiral activity of (rac-MBA)_2_PbI_4_ films became active after the introduction of either one, showing CD spectra similar in magnitude and opposite in sign (Fig. 2F). This implies that equilibrating chiral conformer mixtures can be disturbed by the preferential interaction between (*R*/*S*-MBA)_2_PbI_4_ and (+/−)-TADDOLs. Slightly different coupling strengths in enantiomeric pairs between *R*-MBA: (+)-TADDOL [*S*-MBA: (+)-TADDOL] and *R*-MBA: (−)-TADDOL [*S*-MBA: (−)-TADDOL)] may occur due to a small difference in the degree of steric interaction (*39*), but this requires further in-depth investigation.

### Crystallographic changes in chiral 2D OIHPs by chiral dopants

**Fig. 3.**
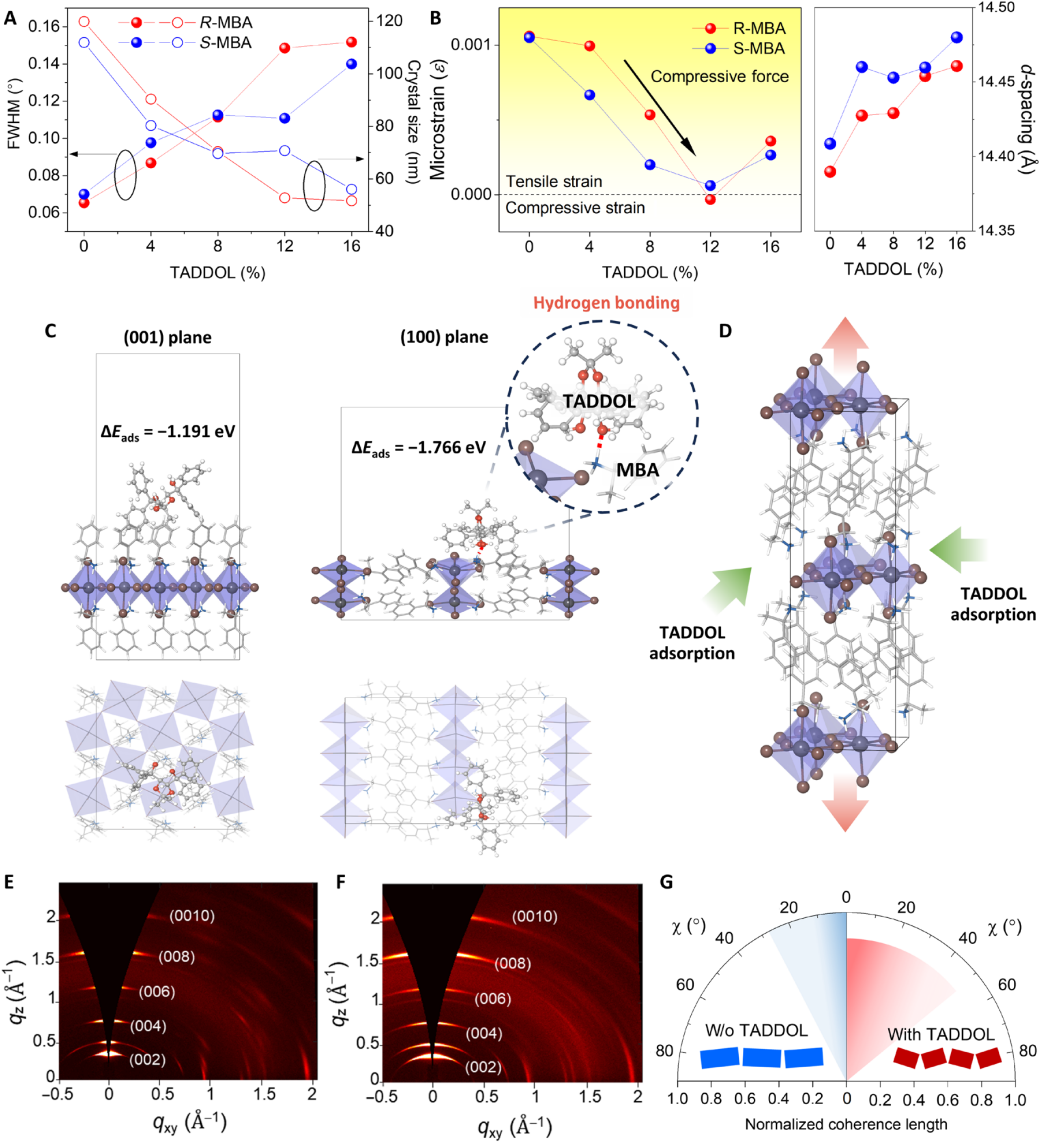
Crystallographic study of chiral 2D OIHPs with TADDOLs. (**A**) Changes in crystal size of chiral 2D OIHPs with the introduction of TADDOLs. (**B**) Changes in microstrain (left) and *d*-spacing (right) of chiral 2D OIHPs with TADDOLs. (**C**) DFT calculations revealed adsorption directions and energies of TADDOLs on chiral 2D OIHPs. (**D**) Lattice expansion configuration in chiral 2D OIHPs induced by biaxial strain from TADDOL adsorption. (**E** and **F**) GWXS patterns and integrated azimuth angle plots of (002) diffraction of (E) the pristine (*R*-MBA)_2_PbI_4_ and (F) TADDOL-introduced (*R*-MBA)_2_PbI_4_ films. (**G**) Schematics displaying the crystal orientation based on integrated azimuth angle plots.

To examine the crystallographic properties of (*R*/*S*-MBA)_2_PbI_4_ influenced by TADDOLs, x-ray diffraction (XRD) analysis was performed (fig. S16). The introduction of TADDOLs led to an increase in the full width at half maximum (FWHM) for the (002) plane peak in XRD, which in turn resulted in a decrease in crystallite size as calculated by the Scherrer equation (Fig. 3A). These crystallographic changes were consistent with the morphological results. The reduction in crystal sizes induced by TADDOLs was also evident in the sharper excitonic peaks observed in absorbance and slight increase in photoluminescence quantum yield, where smaller crystal sizes led to stronger exciton confinement (fig. S17). These results indicate that the crystal growth process of chiral 2D OIHPs was largely constrained by adding TADDOLs.

Next, we examined *d*-spacing and lattice microstrain variance with different concentrations of TADDOLs through Williamson-Hall analysis (Fig. 3B and figs. S18 and S19) (*40*). Notably, continuously increasing trends of *d*-spacing for the (002) plane of (*R*/*S*-MBA)_2_PbI_4_ were observed with TADDOLs. This may have been due to their interactions with chiral organic cations to affect the spatial distance of organic layers (i.e., interlayer distance between inorganic frameworks). This was supported by our x-ray photoelectron spectroscopy results, which showed no noticeable interaction between Pb and TADDOLs (fig. S20). The initial tensile strain of (*R*/*S*-MBA)_2_PbI_4_ was progressively modulated toward compressive strain with the introduction of TADDOLs, which in turn induced octahedral tilting in the inorganic sublattices.

The structural changes in (MBA)_2_PbI_4_ related to applied stress were traced by density functional theory (DFT) calculation (note S2 and figs. S21 and S22A). The biaxial compressive strain applied in the lateral direction (*a*/*b* lattice) induced a tensile strain in the *c* lattice, leading to an increase in *d*-spacing (fig. S22B). Consequently, the Pb-I-Pb angle decreased as the biaxial compressive strain was applied, indicating an increase in in-plane tilting of the inorganic octahedra (fig. S22C). Recent theoretical and experimental investigations have shown that imposing a higher degree of octahedral tilting in chiral 2D OIHPs (i.e., the distortion of Pb-I-Pb bonding angle) can promote chiral transfer by inducing stronger asymmetric hydrogen bonding between the chiral organic cation and inorganic framework (*27*, *34*, *41*, *42*). This suggests that the introduction of TADDOLs not only promotes the long-range organization of chiral 2D OIHPs but also induces octahedral tilting within these structures, both of which contribute to chiral amplification.

To probe the nature of interactions between chiral organic cations and TADDOLs, their adsorption configurations were investigated theoretically (note S2 and figs. S23 to S26). First, we investigated the interaction behavior between the MBA and TADDOL molecules. The amine group of the MBA molecule and the hydroxyl group of the TADDOL molecule demonstrated strong intermolecular hydrogen bond formation, with a binding energy (Δ*E*_bind_) of −2.260 eV (fig. S23A). This insight regarding hydrogen bonding may provide a basis for understanding the driving force behind the coassembly of TADDOLs and chiral 2D OIHPs.

Next, we investigated the surface of the (001) plane (in-plane direction) and the (100) plane (vertical direction) of pristine (MBA)_2_PbI_4_ (fig. S25). Our calculations showed that the surface of the (001) plane was energetically more favorable with lower surface energy of 0.00751 eV Å^−2^ compared to the (100) plane surface (0.00909 eV Å^−2^), which aligns with the [PbI_6_]^4−^ layer oriented parallel to the substrate (*43*). Then, we compared the adsorption energy of TADDOLs on these two planes (Fig. 3C). The (100) plane demonstrated a substantially stronger adsorption energy of −1.766 eV with the TADDOL, while the (001) plane showed an adsorption energy of −1.191 eV with the TADDOL. These observations suggest that TADDOLs find favorable adsorption sites on the (100) plane, as the exposed amine group of MBA facilitates hydrogen bonding with TADDOLs. On the other hand, the (001) plane revealed MBA to be oriented perpendicular to the surface, with the amine group buried and only the phenyl group exposed, thus hindering hydrogen bonding with TADDOLs. The results of DFT calculations corroborated these experimental findings, in which the lateral adsorption of TADDOLs to chiral 2D OIHPs, facilitated by hydrogen bonding between the exposed amine group of MBAs and TADDOLs, culminates in reduction of the crystal size under the influence of applied compressive strain (Fig. 3D).

Coassembly phenomena involving chiral 2D OIHPs and TADDOLs were also conjectured by the DFT calculations, which showed that the binding energy between the MBA and the TADDOL (i.e., −2.260 eV) was twice that between TADDOL molecules (i.e., −1.105 eV) (fig. S23). We further performed the grand canonical Monte Carlo simulations to confirm the feasibility of the prediction based on the molecular interaction strength. The additional TADDOL exhibited a tendency to preferentially adsorb to the amine group of MBA over the TADDOL in the cell (fig. S26). This provides theoretical evidence that the coassembly of TADDOL and MBA molecules is more favorable than strong aggregation of TADDOL molecules among themselves.

To further investigate the impact of TADDOLs on crystallographic properties of chiral 2D OIHPs, grazing-incidence wide-angle x-ray scattering (GIWAXS) patterns were obtained (Fig. 3, E and F, and fig. S27). The crystal orientation of chiral 2D OIHPs became more dynamically disordered after the introduction of TADDOLs, showing broadening of the integrated intensity plots azimuthally along the ring at *q*_r_ assigned to the (002) plane of corresponding films (fig. S28). The azimuth angle for the diffraction signal of the (002) plane was distributed from about −30° to 30°, while it widened from to about −45° to 45° after adding TADDOLs with reduced coherence lengths (Fig. 3G and fig. S29). In contrast to horizontally oriented typical 2D OIHPs based on the continuous plate-like crystal structure in the lateral direction (*44*), the splintered crystals resulting from the penetration of TADDOLs restricted continuous crystal growth in the lateral direction. As a consequence, the splintered crystals exhibited a higher degree of freedom for tilting, contributing to their unique structural characteristics. This facilitated their coassembly with TADDOLs without inducing phase separation.

### Chiral 2D OIHP-based CPL detectors

**Fig. 4.**
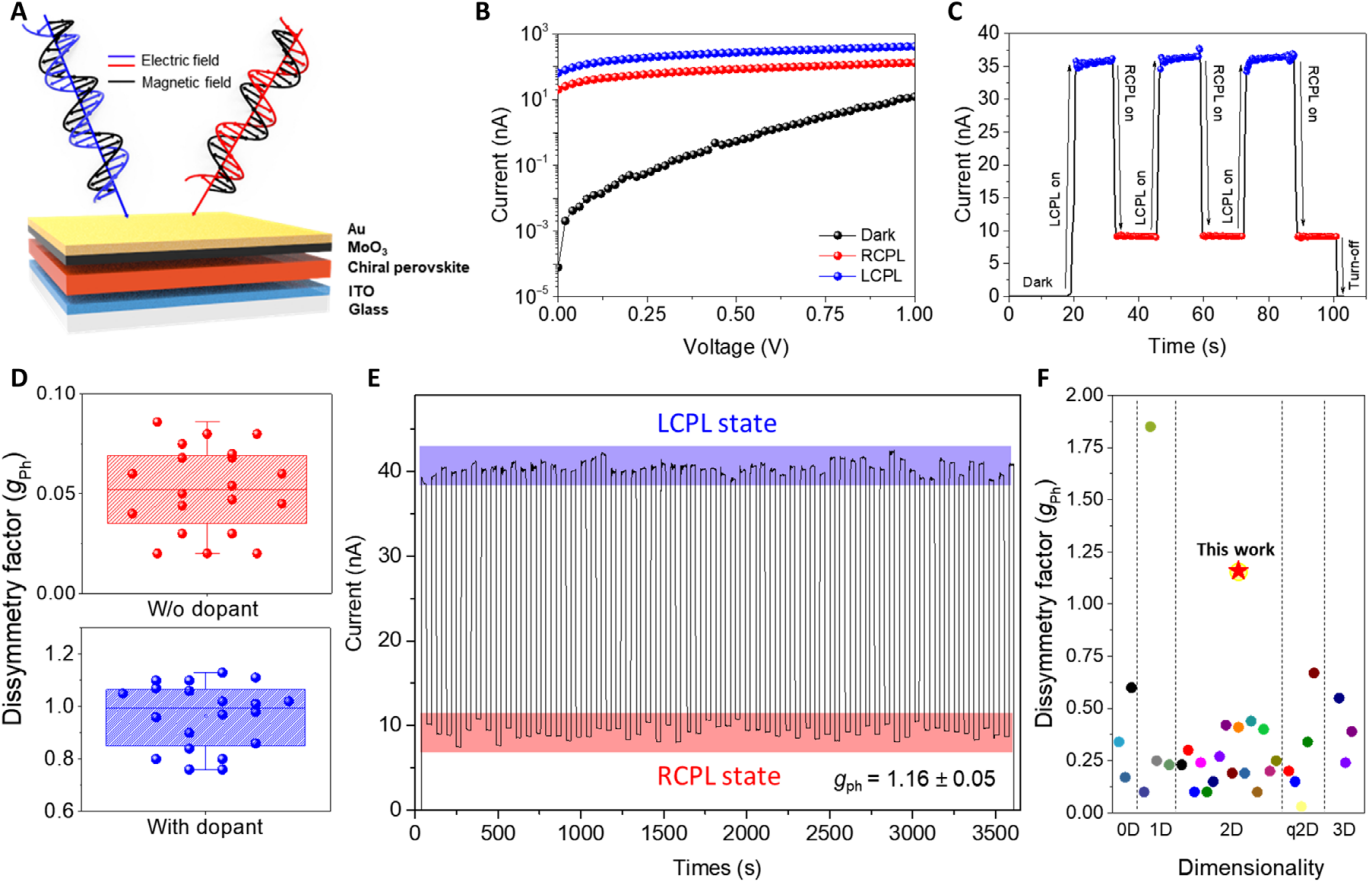
Chiral 2D OIHP-based CPL detectors. (**A**) Device configuration. (**B**) *I*-*V* characteristics under modulation of the SAM of the light. (**C**) Time-dependent detection characteristics. (**D**) Statistics of *g*_Ph_ obtained from optimized 20 devices for each chiral 2D OIHP film. (**E**) Photocurrent monitoring during stepwise modulation of the SAM of the incident beam for 1 hour. (**F**) Comparison of this work with previous studies in terms of *g*_Ph_ and dimensionality (see table S3).

To demonstrate the effectiveness of chiral amplification in electronic devices, we fabricated a CPL detector with the device configuration: indium tin oxide (ITO)/chiral 2D OIHPs/MoO_3_/Au (Fig. 4A). The photocurrent characteristics under steady-state and real-time modes at zero bias demonstrated that polarization-dependent photon absorption resulted in notable differences in photocurrents depending on the SAM of the incident light at a wavelength of 491 nm (Fig. 4, B and C). The incorporation of chiral dopants endowed our system with a robust circular polarization detection ability at zero bias, yielding a remarkable *g*_Ph_ exceeding 1.1, which was also accompanied by notably enhanced specific detectivity (D*), photoresponsivity (*R*), and external quantum efficiency compared to the pristine device (figs. S30 and S31). TADDOL-introduced CPL detector also verified faster response time and higher linear dynamic range compared to the pristine device (figs. S32 and S33). Figure 4D reveals the higher and reproducible *g*_Ph_ afforded by the chiral dopant. The accuracy and reproducibility of SAM detection were further confirmed by monitoring photocurrent as a function of time during real-time stepwise modulation of the SAM of the incident beam for 1 hour, with an average *g*_Ph_ of 1.16 (Fig. 4E). The observed *g*_Ph_ value far exceeded the state-of-the-art performance for chiral 2D OIHP-based CPL detectors (Fig. 4F and table S3), establishing an excellent correlation with the giant chiral amplification effect in absorbance.

## DISCUSSION

We achieved highly amplified chiroptical activity in typical chiral 2D OIHPs through chiral dopant-assisted dynamic crystal reconstruction, which enabled strengthened lattice distortion and intriguing morphological evolution with a long-range chiral order. We found that the strong adsorption of chiral dopants to chiral organic cations plays a key role in inducing dynamic crystal reconstruction. Chiral 2D OIHP films with chiral dopants enabled highly sensitive discrimination of CPL with a greatly amplified *g*_CD_, outperforming the limited chiroptical activity of typical MCIFs. The resulting CPL detectors exhibited remarkable sensitivity for SAM of incident photons in real time with *g*_Ph_ above 1.0 under zero bias, which is the highest value reported to date for chiral 2D OIHP-based CPL detectors. In contrast to existing MCIFs, our results provide a powerful tool for designing efficient chiral 2D OIHPs, thereby having a strong influence on the anisotropic behavior in chiral optoelectronics. Our findings will accelerate the development of advanced quantum cryptography and optical communication using the spin state control of light.

## MATERIALS AND METHODS

### Materials

(*R*)-(+)-α-methylbenzylamine (*R*-MBA, 98%, optical purity > 97%), (*S*)-(−)-α-methylbenzylamine (*S*-MBA, 98%, optical purity > 97%), *N*,*N*-anhydrous dimethylformamide (DMF), anhydrous dimethyl sulfoxide (DMSO), lead oxide (PbO, 99.999%), and 57% aqueous hydriodic acid (HI) solution (99.95%, stabilized by H_3_PO_2_) were purchased from Sigma-Aldrich (St. Louis, MO, USA). Acetone (99.8%) and isopropanol (99.5%) were purchased from Daejung Chemical (Shiheung, Korea). (2*S*,3*S*/2*R*,3*R*)-2,3-*O*-isopropylidene-1,1,4,4-tetraphenyl-1,2,3,4-butanetetrol, (+/−)-TADDOLs were purchased from TCI Chemicals (Tokyo, Japan).

### Fabrication of chiral 2D OIHP films and CPL detectors

An amount of 0.5 g (2.2 mmol) PbO was fully dissolved in 3 ml of HI solution (57 wt %) and heated to 140°C with stirring at 1000 rpm. Then, 570 μl of *R*- or *S*-MBA was reacted with 728 μl of HI solution in an ice bath for 20 min and added to this PbO/HI solution. The mixture was allowed to cool to room temperature under ambient conditions, resulting in the formation of orange crystals. The crystals were vacuum-filtrated, and the filtrate was rinsed with hexane several times. Last, the product was dried in a vacuum oven overnight. Then, the films were spin-coated on ITO glass substrates cleaned by sonication in deionized water/acetone/isopropanol for 10 min each and using an ultraviolet/ozone cleaner for 15 min. To prepare chiral 2D OIHP films, 60 μl of 0.3 M perovskite solution in DMF containing (+/−)-TADDOL at different molar ratios to chiral cations was spread on the ITO glass substrate, and spin coating was performed at 4000 rpm for 30 s. The spin-coated film was annealed at 100°C for 10 min on a hotplate. To fabricate vertically configured CPL detectors, chiral 2D OIHP polycrystalline film was fabricated by spin coating in the same way as described above on ITO glass. Then, a 4-nm-thick layer of MoO_3_ and 20-nm-thick layer of Au were thermally evaporated under vacuum (<10^−6^ mbar) in sequence to form an electrode (active area of 200 μm by 200 μm).

### Film and device characterization

XRD analyses were conducted using an x-ray diffractometer (Smart Lab, Rigaku, Tokyo, Japan) with Cu Kα radiation (λ = 1.5406 Å) at 40 kV/40 mA. SEM was performed using a field emission SEM (JSM-7800F Prime; JEOL, Tokyo, Japan). TEM images were acquired using a JEM-ARM200F (JEOL) electron microscope installed at the National Center for Inter-university Research Facility at Seoul National University. The microscope was equipped with a cold emission gun, a spherical aberration corrector at image-forming lens, and a direct electron detector (K3-IS; Gatan, Pleasanton, CA, USA). TEM imaging was performed with an electron dose rate of 10 to 20 e^−^ Å^−2^ s^−1^ to alleviate structural degradation of the sample. GIWAXS measurements were conducted on the PLS-II 9A U-SAXS beamline at the Pohang Accelerator Laboratory in Korea. X-rays emitted from the in-vacuum undulator were monochromated at 11.025 keV (λ = 1.12454 Å) using a double-crystal monochromator and then focused both horizontally and vertically [450 μm (H) by 60 μm (V) in the FWHM sample position] using K-B–type mirrors. The sample stage was equipped with a seven-axis motorized stage for fine alignment of the sample, and the incidence angle of the x-ray beam was set to a range of 0.12° for structural analysis, which was close to the critical angle of the samples. The patterns were recorded with a 2D charge-coupled device detector (SX165; Rayonix, Evanston, IL, USA); the x-ray irradiation time ranged from 30 to 60 s according to the saturation level of the detector. Diffraction angles were calibrated using precalibrated sucrose (monoclinic, *P*2_1_, *a* = 10.8631 Å, *b* = 8.7044 Å, *c* = 7.7624 Å, β = 102.938°), and the sample-to-detector distance was ~220.8 mm. Absorption spectra were measured using an ultraviolet/visible light spectrophotometer (V-770; Jasco, Tokyo, Japan). *I*–*V* and *I*–*t* curves were measured under vacuum using a parametric analyzer (4200-SCS; Keithley, Solon, OH, USA). CPL illumination was generated using a linear polarizer and a quarter-wave plate (Thorlabs, Newton, NJ, USA) installed between the light source and sample. The CPL direction of the laser light was controlled by wave plate rotation. CD spectroscopy was performed using a CD spectropolarimeter (J-815; Jasco). The *g*_CD_, indicating the ratio of CD to conventional absorption, was calculated using the following equation

$$g_{\text{CD}}=2\frac{A_{L}-A_{R}}{A_{L}+A_{R}}=\frac{\text{CD}}{32980\times A}$$

where *A*_L_ is the absorption for LCPL, *A*_R_ is the absorption for RCPL, CD is the value extracted from CD spectroscopy, and *A* is the absorbance of the sample. The *g*_PL_ was defined as

$$g_{\text{PL}}=2\frac{I_{\text{PL},L}-I_{\text{PL},R}}{I_{\text{PL},L}+I_{\text{PL},R}}$$

where *I*_PL,L_ and *I*_PL,R_ indicate the photoluminescence emission intensity of LCPL and RCPL, respectively. The discrimination ability for CPL light of CPL detectors was characterized by *g*_Ph_ in analogy to *g*_CD_ as follows

$$g_{\text{Ph}}=\frac{2\left(I_{\text{Ph},L}-I_{\text{Ph},R}\right)}{\left(I_{\text{Ph},L}+I_{\text{Ph},R}\right)}$$

where *I*_Ph,L_ and *I*_Ph,R_ are the photocurrents under LCPL and RCPL illumination, respectively.

## Supplementary Materials

### This PDF file includes:

Supplementary Notes S1 and S2 Figs. S1 to S33 Tables S1 to S3 References
