# Supplementary material for: Giant chiral amplification of chiral 2D perovskites via dynamic crystal reconstruction
Source: Sci Adv. 2024 Aug 21;10(34):eado5942. doi: 10.1126/sciadv.ado5942 (PMC11338236; doi:10.1126/sciadv.ado5942)
Supplement: Supplementary file 1 — Supplementary Notes S1 and S2 Figs. S1 to S33 Tables S1 to S3 References [file sciadv.ado5942_sm.pdf]

Science Advances
AAAS

Supplementary Materials for

Giant chiral amplification of chiral 2D perovskites via dynamic crystal reconstruction

Hongki Kim *et al.*

Corresponding author: Joon Hak Oh, joonhoh@snu.ac.kr; Sang Kyu Kwak, skkwak@korea.ac.kr

Sci. Adv. **10**, eado5942 (2024)
DOI: 10.1126/sciadv.ado5942

This PDF file includes:

Supplementary Notes S1 and S2
Figs. S1 to S33
Tables S1 to S3
References

Note S1. Estimation of *m* and R_{eg}

The dissymmetry factor g_{PL} relates to μ and m of the optical transition by the following equation:

$$g_{PL} = \frac{4R_{eg}}{m^2+\mu^2} = \frac{4m\mu\ cos\theta}{m^2+\mu^2} \qquad \text{Eq. S1}$$

where m and μ are the magnetic and electric dipole moments, respectively, and θ is the angle between the dipole moments. R_{eg} represents rotational strength, which is directly proportional to g_{PL} and the determination of g_{PL} requires non-vanishing R_{eg} (*1*). The estimation of m was performed by calculating μ with the measured g_{PL} value using a circularly polarized luminescence spectrophotometer (Fig. S14). To calculate μ, the radiative recombination rate (k_{rad}) of the transition was estimated using the following equation (*2*):

$$k_{rad} = \frac{\omega^3 n^3 \mu^2}{3\pi\varepsilon_0 \hbar c^3} \qquad \text{Eq. S2}$$

where ω is the frequency of wavelength, n is the refractive index of the perovskite (set to 2.3 as a typical value based on a previous report) (*3*), $\hbar$ is the reduced plank constant, ε_0 is the vacuum permittivity, and c is the speed of light in a vacuum. k_{rad} was obtained using the following equation:

$$PLQY = \frac{k_{rad}}{k_{measured}} = \frac{k_{rad}}{k_{rad}+k_{non-rad}} \qquad \text{Eq. S3}$$

where PLQY is the photoluminescence quantum yield of the chiral 2D OIHP film, which was obtained from steady-state PL study (Fig. S17), $k_{non\text{-}rad}$ is the nonradiative decay rate, and $k_{measured} = k_{rad} + k_{non\text{-}rad}$ is the total decay rate obtained from time-resolved photoluminescence (TRPL) as it is equal to the reciprocal of lifetime ($1/\tau$) (Fig. S8). From TRPL study and obtained PLQY, we calculated the k_{rad}, which enabled the estimation of μ. Then, using the obtained g_{PL} and μ, and setting θ to be 0° in estimations for convenience, we calculated the m and R_{eg}.

Note S2. Calculation details

2–1. Investigation of structural changes induced by biaxial strain in $(MBA)_2PbI_4$

We performed spin-polarized DFT calculations on the bulk and surface structures of $(MBA)_2PbI_4$ perovskite using the Vienna ab initio simulation package (VASP) (*4*, *5*). In these calculations, the exchange-correlation potential of electrons was represented by the generalized gradient approximation (GGA), in conjunction with the Perdew-Burke-Ernzerhof functional (PBE) (*6*). The interaction between electrons and ions were portrayed utilizing the projector-augmented-wave (PAW) method (*7*). Atomic positions were fully relaxed until the Hellmann-Feynman force converged to less than −0.02 eV $Å^{-1}$. We set a convergence criterion of 1×10^{-6} eV for the change in energy within the self-consistent field calculations. The DFT-D3 dispersion correction method developed by Grimme et al. was employed to account for the long-range van der Waals interaction (*8*, *9*). For the sampling of the Brillouin zone, we utilized a gamma-centered Monkhorst-Pack *k*-point grid: specifically, a 5 × 5 × 1 *k*-point grid for bulk structures, and a gamma point for surface structures (*10*). Moreover, dipole slab corrections were incorporated for the surface systems.

The structural optimization process of $(MBA)_2PbI_4$ perovskite involved a complete relaxation of both lattice parameters and atomic positions (*11*). The optimized bulk structure possesses the $P2_12_12_1$ space group, with lattice parameters of a = 9.18 Å, b = 8.83 Å, and c = 28.61 Å, with $\alpha = \beta = \gamma = 90°$ (Fig. S21). A calculated *d*-spacing of 14.31 Å aligns closely with the experimental value of 14.4 Å, suggesting an error of approximately 0.6%.

We applied biaxial strain in the lateral direction to investigate structural changes in the $(MBA)_2PbI_4$ perovskite (Fig. S22) (*8*). In the DFT calculations, we decreased the value of strain (ε) from 0% (no strain) to −6% (compressive) in 1% increments. The biaxial strain was simulated by modifying the lattice parameters a and b according to the equation $a = a_0 (1 + \varepsilon)$, $b = b_0 (1 + \varepsilon)$, where a_0 and b_0 are the original lattice constants. The tensile strain induced in the c lattice was calculated using the equation $c = c_0 (1 + \varepsilon)$, with c_0 representing the original lattice constant.

2–2. Binding energy calculation for MBA and TADDOL molecules

Grand canonical Monte Carlo (GCMC) simulation and density functional theory (DFT) calculation were used to calculate the binding energies of TADDOL and MBA molecules. To uncover the preferential binding configurations, we performed GCMC simulations using the

Sorption program (*12*). The interatomic interactions were portrayed using the COMPASSII forcefield (*13*). Non-bond interactions, which include electrostatic and van der Waals forces, were estimated using the Ewald summation and an atom-based cutoff scheme (a radius of 15.5 Å), respectively. The GCMC simulation proceeded with 1.0×10^6 steps for production employing the configurational bias algorithm.

The DMol^3 program was used to calculate the binding energies of TADDOL and MBA molecules via DFT calculations (*14*). The generalized gradient approximation (GGA) paired with the Perdew–Burke–Ernzerhof (PBE) functional was used to illustrate the exchange-correlation potential (*6*). We performed dispersion correction of van der Waals effects using the Grimme scheme (*15*). All electronic relativistic effects were accommodated in our calculations, which involved the treatment of core electrons. A global orbital cutoff radius of 3.7 Å was used for this purpose. We adopted the double numerical basis set with polarization functions (version 4.4) in our calculations. Convergence criteria were established as follows: energy convergence at 1.0×10^{-5} Hartree (Ha), displacement convergence at 5×10^{-3} Å, and force convergence at 2×10^{-3} Ha Å^{-1}.

Our DFT calculations were performed for *S*-MBA and (−)-TADDOL. The binding energy of these molecules was evaluated using Eq. S4:

$$\Delta E_{\mathrm{bind}} = E_{\mathrm{MBA+TADDOL}} - E_{\mathrm{MBA}} - E_{\mathrm{TADDOL}} \qquad \text{Eq. S4}$$

where $E_{\mathrm{MBA+TADDOL}}$, E_{MBA}, and E_{TADDOL} represent the total energy of the system when MBA and TADDOL interact, and the total energies of individual MBA and TADDOL molecules, respectively. The binding structure obtained via GCMC simulations demonstrated that a hydrogen bond forms between MBA and TADDOL, presenting a binding energy of −2.260 eV (Fig. S23A). This hydrogen bonding interaction was found to be stronger than π–π interactions (Fig. S24), underscoring the significant role of hydrogen bonding in the interaction between MBA and TADDOL. Furthermore, we calculated the interaction between two TADDOL molecules using Eq. S5:

$$\Delta E_{\mathrm{bind}} = E_{\mathrm{TADDOL+TADDOL}} - 2E_{\mathrm{TADDOL}} \qquad \text{Eq. S5}$$

where $E_{\mathrm{TADDOL+TADDOL}}$ and E_{TADDOL} represent the total energy of the structure in which two TADDOL molecules interact, and the total energy of a single TADDOL molecule, respectively.

2–3. Surface energy and TADDOL adsorption energy calculation on the $(MBA)_2PbI_4$ surface

We constructed (001) and (100) surfaces by including a 20 Å vacuum slab in the *z*-direction to preclude self-interactions within the slab (Fig. S25) (*16*). The atomic positions of the lower half of the surface layer were held constant during these calculations. The initial adsorption positions of the TADDOL molecule on the $(MBA)_2PbI_4$ surface were determined via GCMC simulations. For these simulations, we utilized the Universal force field and Mulliken charges derived from DFT calculations (*17*, *18*). The surface energy of the $(MBA)_2PbI_4$ perovskite was computed using Eq. S6:

$$\gamma = (E_S - nE_b)/2A \qquad \text{Eq. S6}$$

In this equation, E_S, E_b, n, and A represent the energy of the surface model, the bulk energy of the unit cell, the number of total formula units of the surface model, and the surface area, respectively. Note that the (001) plane was energetically more favorable as its surface energy was 0.00751 eV Å^{-2}, while that of the (100) plane was 0.00909 eV Å^{-2}. The lower surface energy of the (001) plane could be ascribed to the absence of bond-breaking, thereby preserving the sixfold coordination of Pb (*16*). On the other hand, the (100) plane demonstrated a higher surface energy as the Pb atomic coordination was reduced, leading to the formation of truncated PbI_6 octahedra with exposed Pb atoms (i.e., PbI_6 to PbI_5).

We also calculated the adsorption energies of TADDOL on the $(MBA)_2PbI_4$ surface as follows (Eq. S7):

$$\Delta E_{ads} = E_{MBA_2PbI_4surface+TADDOL} - E_{MBA_2PbI_4surface} - E_{TADDOL} \quad \text{Eq. S7}$$

where $E_{MBA_2PbI_4surface+TADDOL}$, $E_{MBA_2PbI_4surface}$, and E_{TADDOL} represent the total energies of the $(MBA)_2PbI_4$ surface when interacting with a TADDOL molecule, the $(MBA)_2PbI_4$ surface system, and the TADDOL molecule, respectively.

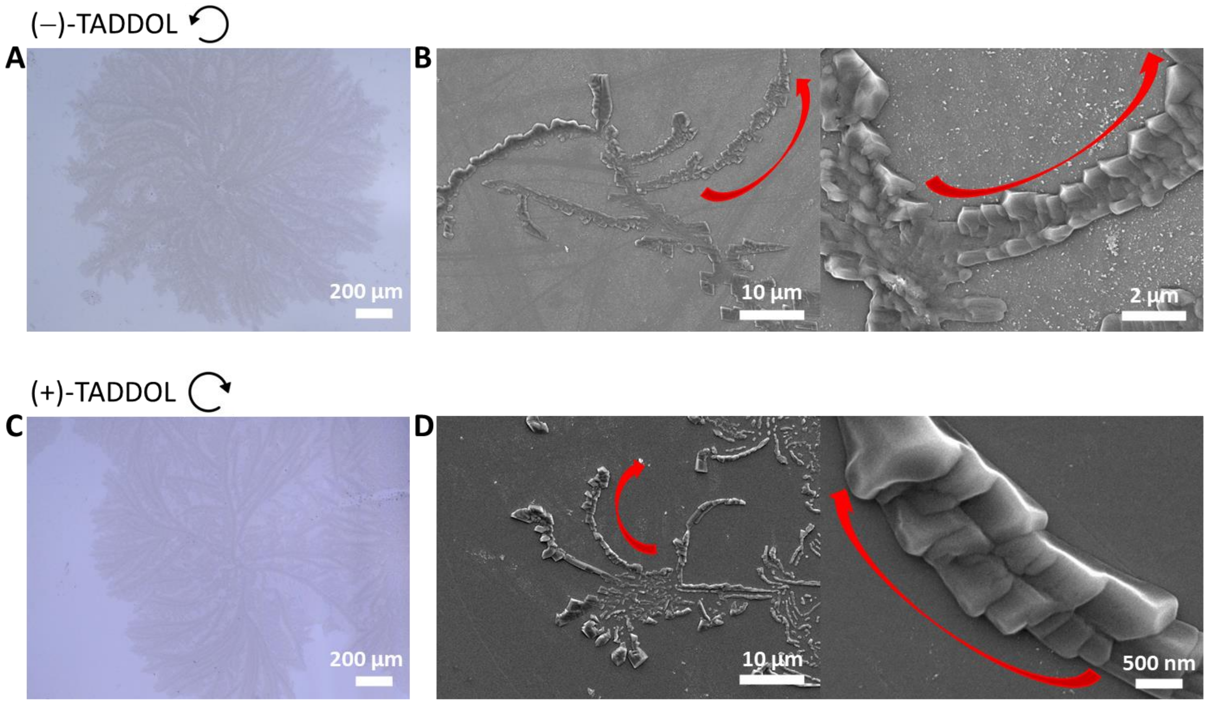

Fig. S1. Morphological analysis of TADDOL films. (A) Optical microscopy image of (−)-TADDOL films and **(B)** their magnified morphologies in SEM. **(C)** Optical microscopy image of (+)-TADDOL films and **(D)** their magnified morphologies in SEM. Different chiral enantiomers produce opposite chiral handedness, enabling long-range ordered chiral assemblies. TADDOL films were fabricated by spin-coating the TADDOL solution at a concentration of 10 mg mL^{-1} in DMF, followed by annealing at 100°C for 10 min.

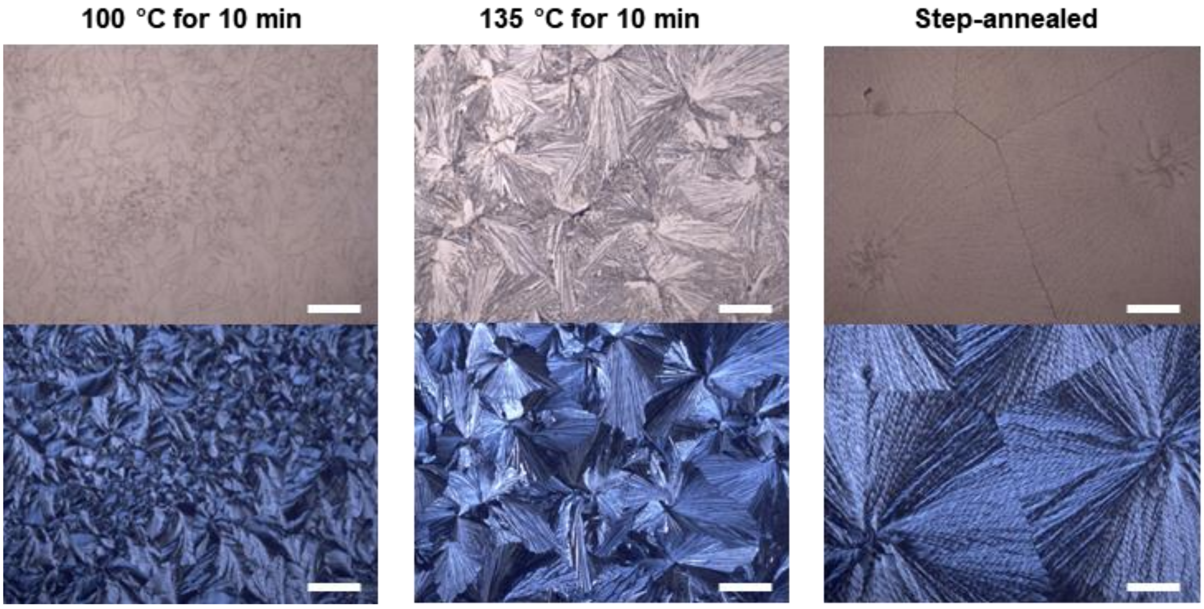

Fig. S2. Morphological analysis of TADDOL films with different annealing conditions. Nonpolarized optical microscopy images of TADDOL films (top) corresponding microscopy images under cross-polarized filters (down), with different annealing conditions: annealing at 100°C for 10 min (left), annealing at 135°C for 10 min (middle), annealing at 80°C increased by 5°C per min up to 135°C, then annealed for 15 min at 135°C (step-annealing) (right). Inset scale bars represent 300 µm. TADDOL films exhibited unique morphologies depending on the processing conditions, and their spherulite-like morphologies originated from their preferred supramolecular assemblies. Processing conditions presumably affected the assembly properties of TADDOLs, resulting in differences in homogeneity and domain size.

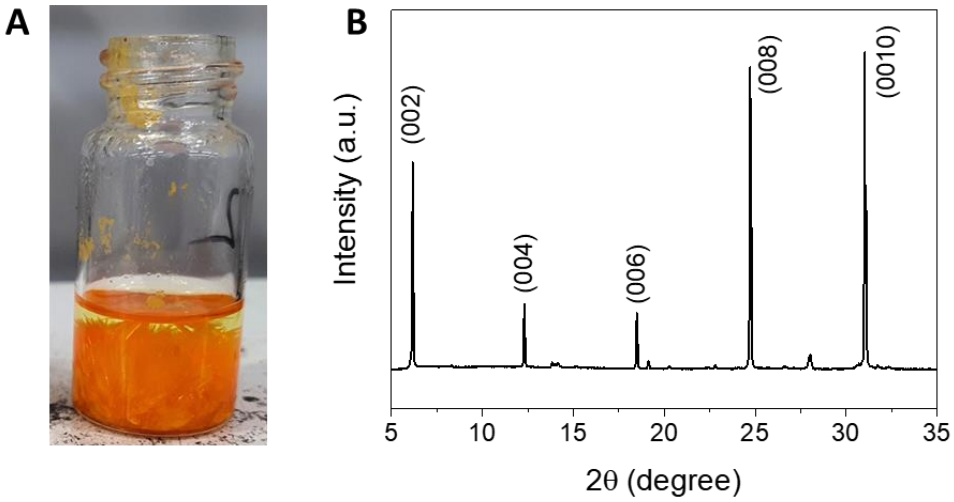

Fig. S3. Material verification of chiral 2D OIHPs. (A) Photograph of a synthesized single crystal of chiral 2D OIHP, and **(B)** corresponding XRD diffractogram.

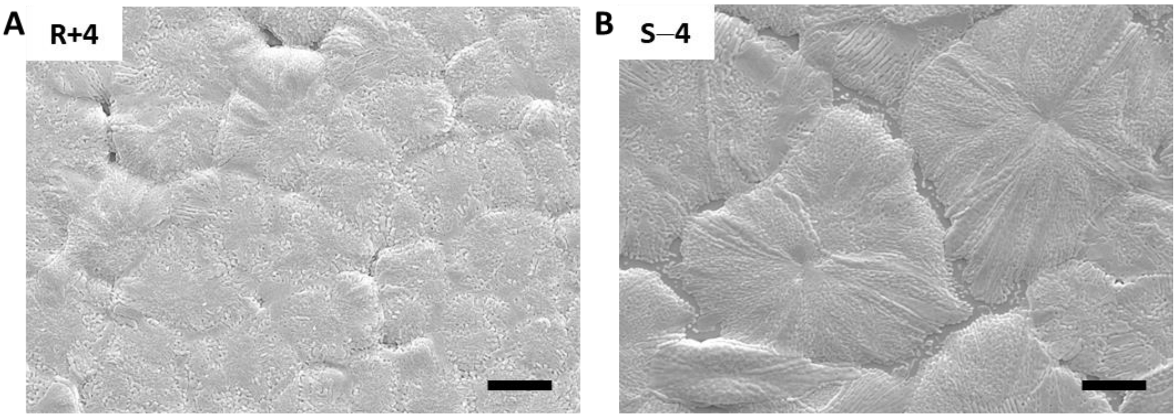

Fig. S4. SEM images of TADDOL-introduced chiral 2D OIHP films. (A) (+)-TADDOL (4%)-introduced (*R*-MBA)$_2$PbI$_4$ (R+4), **(B)** (−)-TADDOL (4%)-introduced (*S*-MBA)$_2$PbI$_4$ films (S–4). Inset scale bars in SEM images represent 2 μm.

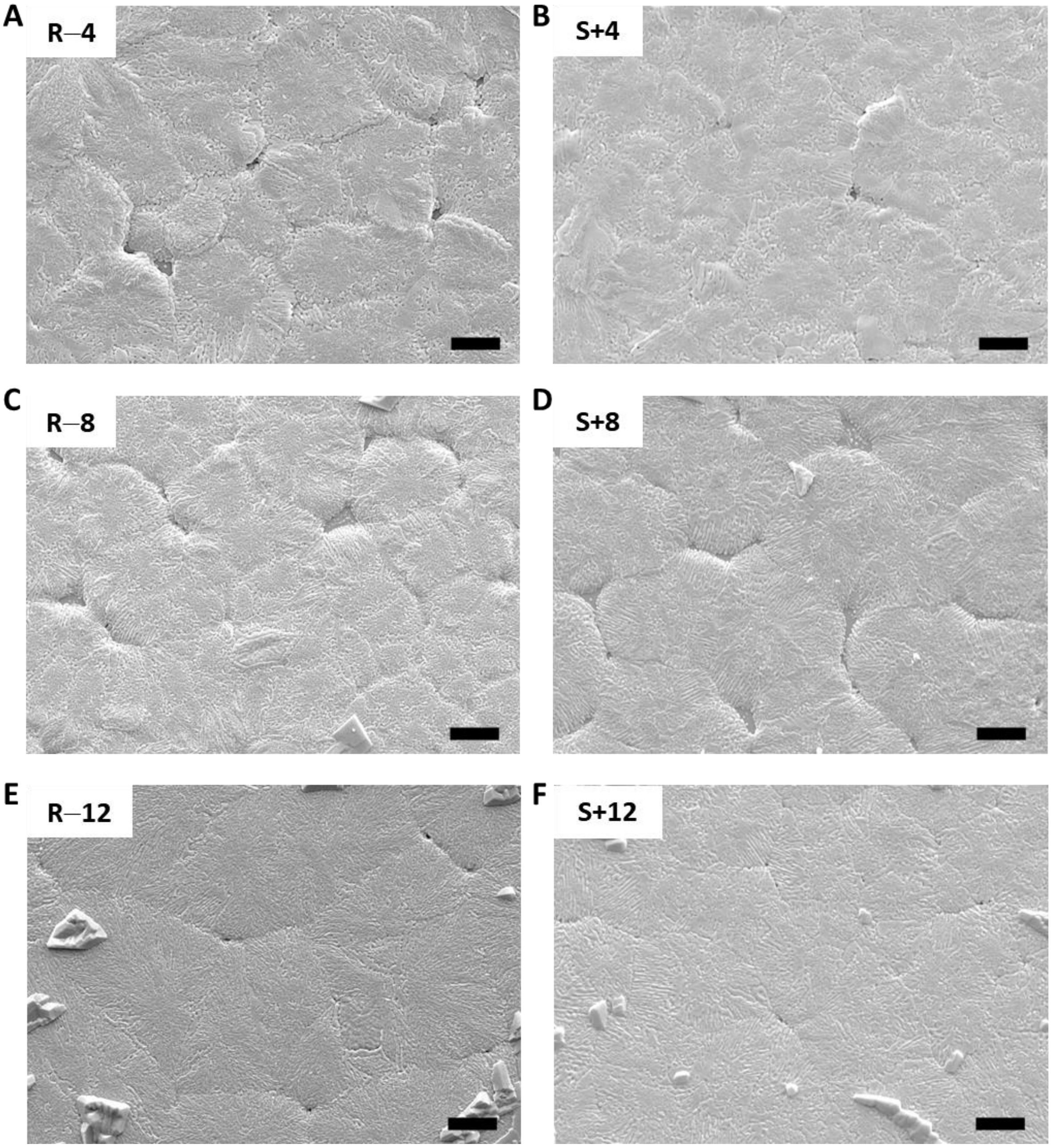

Fig. S5. SEM images of TADDOL-introduced chiral 2D OIHP films. (A) (−)-TADDOL (4%)-introduced (*R*-MBA)$_2$PbI$_4$ (R–4), **(B)** (+)-TADDOL (4%)-introduced (*S*-MBA)$_2$PbI$_4$ films (S+4), **(C)** (−)-TADDOL (8%)-introduced (*R*-MBA)$_2$PbI$_4$ (R–8), **(D)** (+)-TADDOL (8%)-introduced (*S*-MBA)$_2$PbI$_4$ films (S+8), **(E)** (−)-TADDOL (12%)-introduced (*R*-MBA)$_2$PbI$_4$ (R–12), **(F)** (+)-TADDOL (12%)-introduced (*S*-MBA)$_2$PbI$_4$ films (S+12). Inset scale bars in SEM images represent 2 μm.

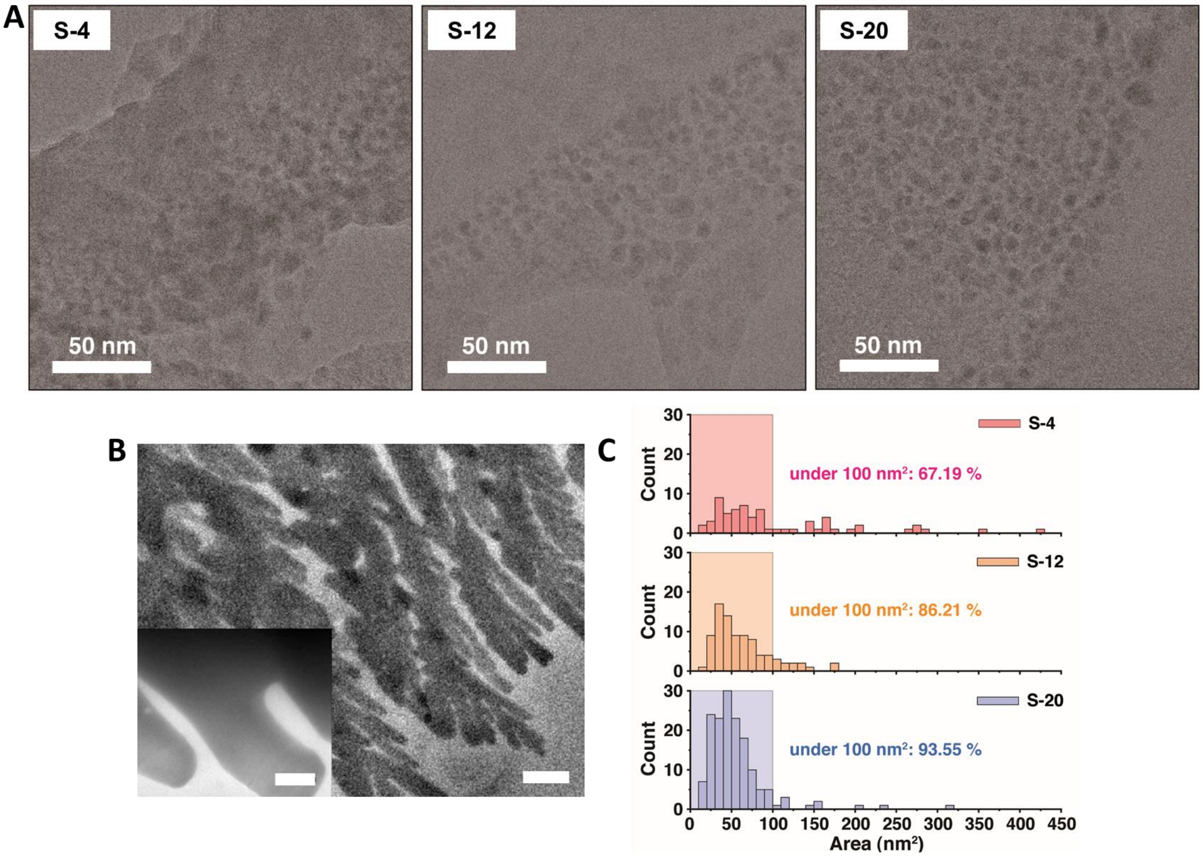

Fig. S6. TEM of TADDOL-introduced chiral 2D OIHP films. (A) TEM images for the (*S*-MBA)$_2$PbI$_4$ films with 4% (−)-TADDOL (S–4), 12% (−)-TADDOL (S–12), and 20% (−)-TADDOL (S–20). **(B)** TEM image of chiral 2D OIHPs with a TADDOL (scale bar = 0.2 μm). Inset TEM image shows pristine chiral 2D OIHP (scale bar = 1 μm). **(C)** Changes in crystal size of chiral 2D OIHPs with the introduction of TADDOLs.

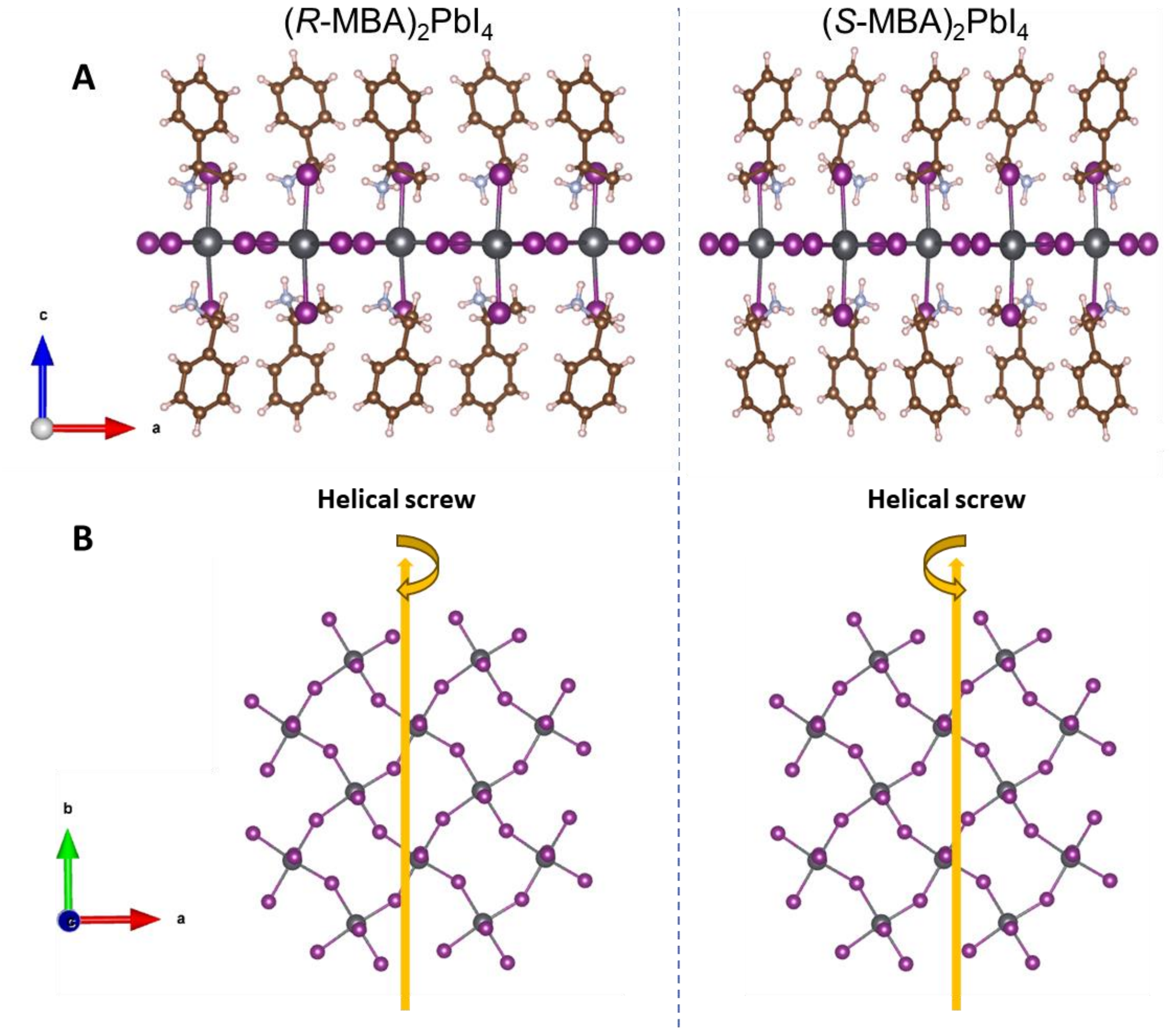

Fig. S7. Schematic X-ray single-crystal structures of (*R*-MBA)$_2$PbI$_4$ and (*S*-MBA)$_2$PbI$_4$. (A) In-plane views of $[PbI_4]^{2-}$ layers in (*R*-MBA)$_2$PbI$_4$ (left) and (*S*-MBA)$_2$PbI$_4$ (right). **(B)** Halide helical screw of purple I atoms is shown from the Pb–I–Pb bonds indicated by yellow arrows.

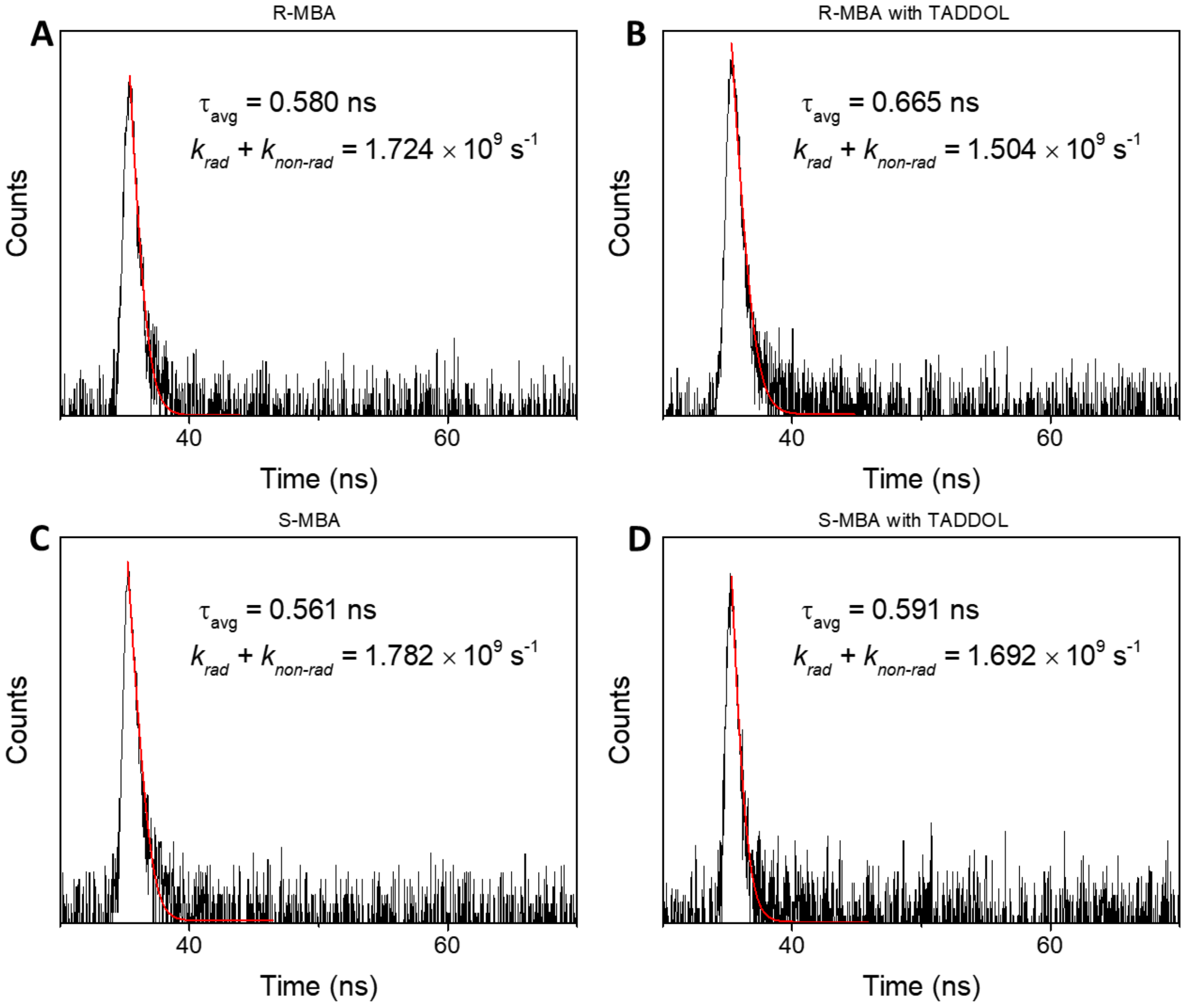

Fig. S8. Time-resolved photoluminescence (TRPL) measurements. (A to **D)**, Decay curves of (R-MBA)$_2$PbI$_4$ films (A) without TADDOLs or (B) with TADDOLs, and the (S-MBA)$_2$PbI$_4$ films (C) without TADDOLs or (D) with TADDOLs.

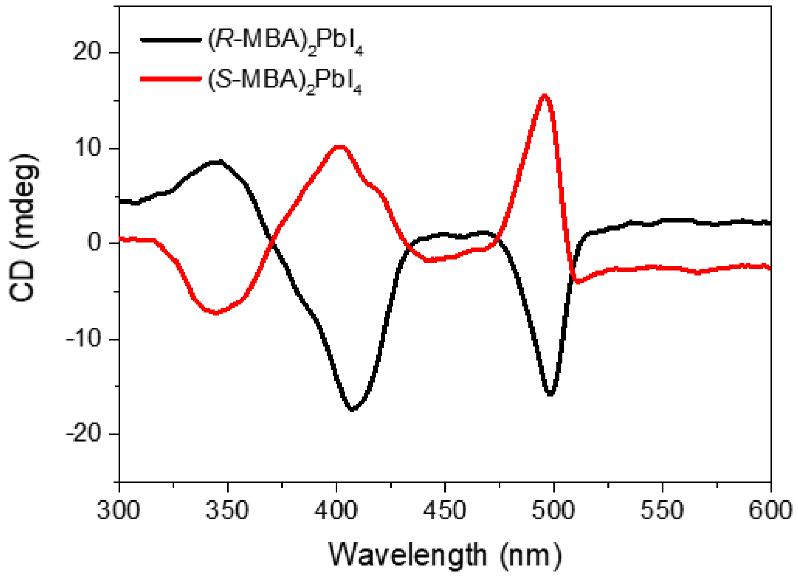

Fig. S9. CD spectra of pristine (*R*/*S*-MBA)$_2$PbI$_4$ films.

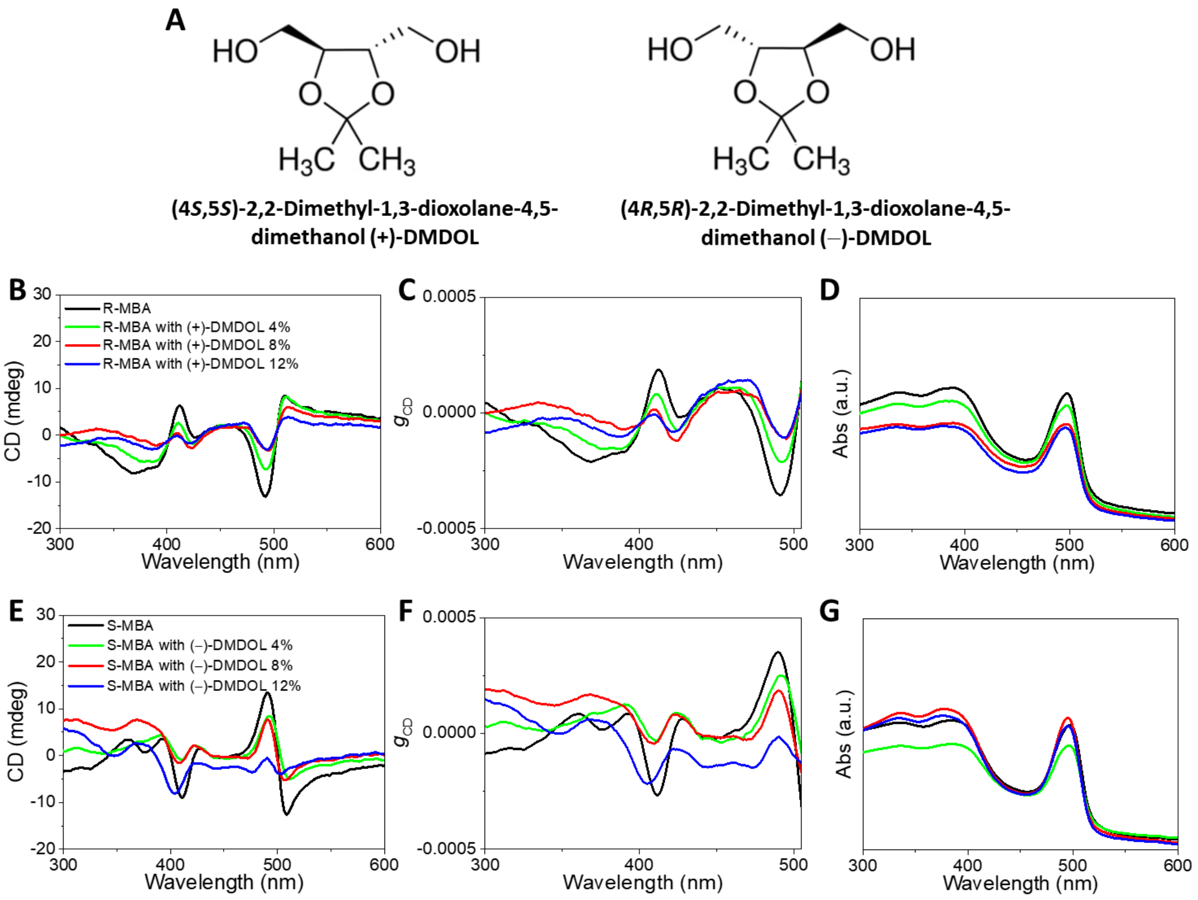

Fig. S10. Control experiments using chiral molecules ((+)-DMDOL or (−)-DMDOL) with the same structure with TADDOLs except that lacking benzene rings. (A) Molecular structures of (+)-DMDOL and (−)-DMDOL. **(B)** CD spectra, **(C)** g_{CD}, **(D)** absorption spectra of (R-MBA)$_2$PbI$_4$ films with the introduction of different amounts of (+)-DMDOLs. **(E)** CD spectra, **(F)** g_{CD}, **(G)** absorption spectra of (S-MBA)$_2$PbI$_4$ films with the introduction of different amounts of (−)-DMDOLs.

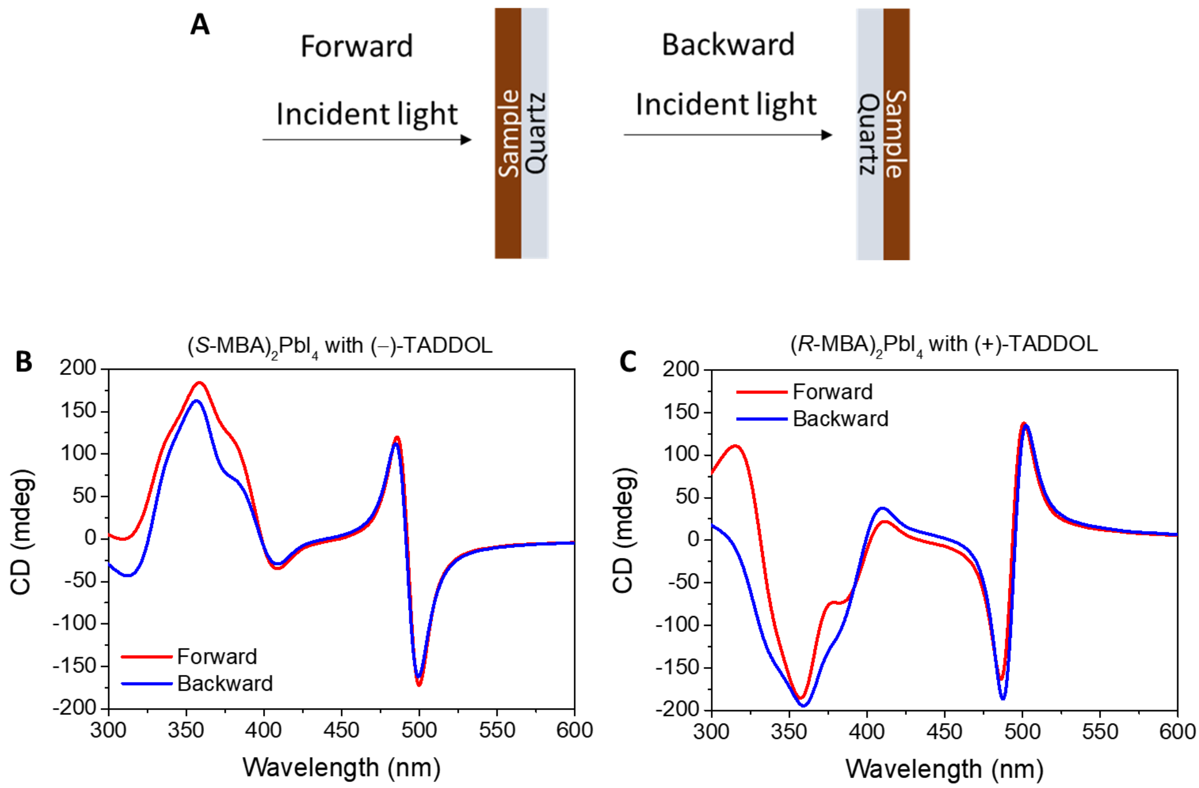

Fig. S11. LDLB effect of TADDOL-introduced chiral OIHP films. (A) CD measurements from the front (forward) and the back side (backward) of the thin film, **(B)** CD spectra of (*S*-MBA)$_2$PbI$_4$ with (−)-TADDOL recorded from the front (forward) and the back side (backward) of the thin film, **(C)** CD spectra of (*R*-MBA)$_2$PbI$_4$ with (+)-TADDOL recorded from the front (forward) and the back side (backward) of the thin film.

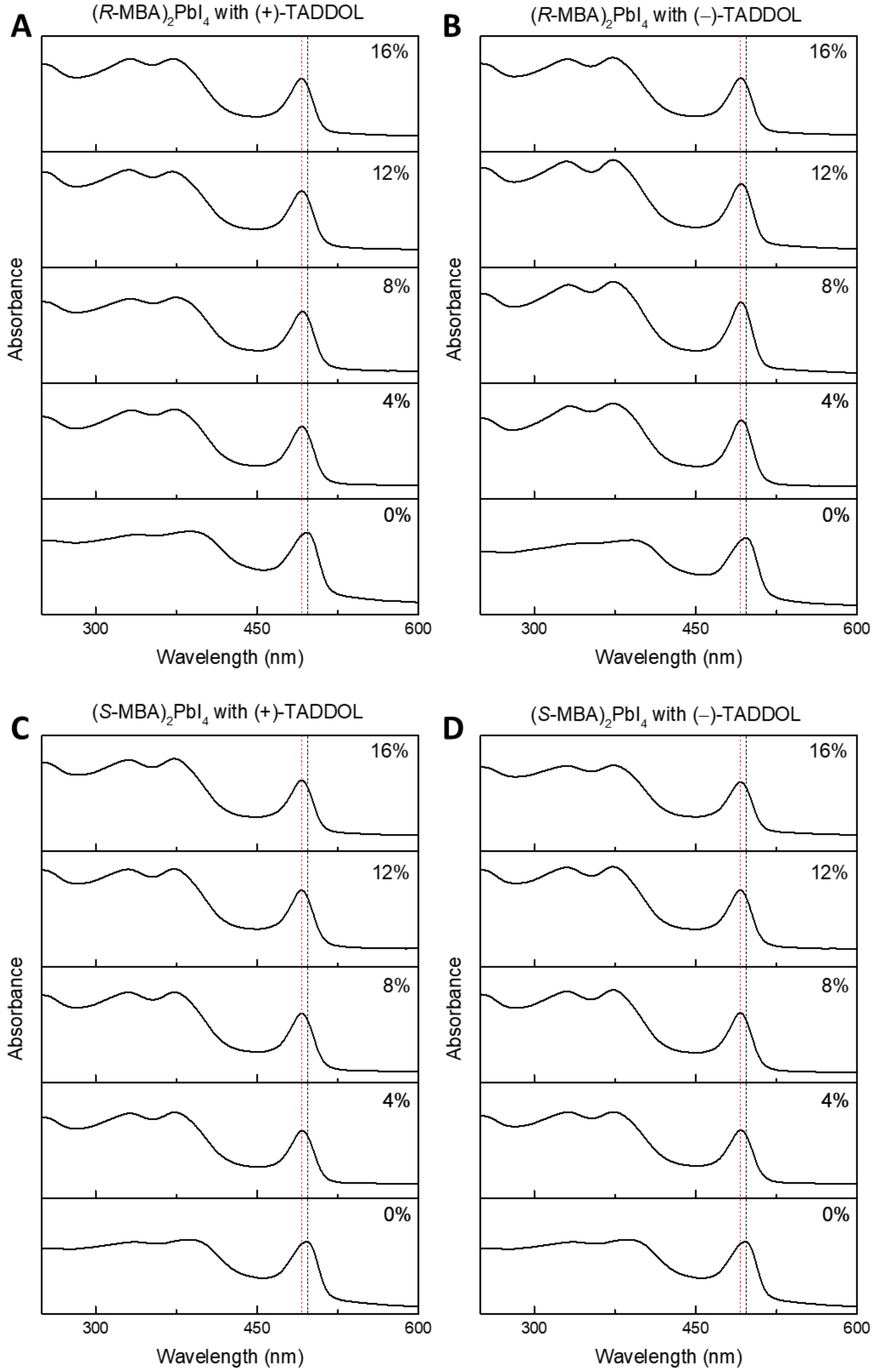

Fig. S12. Absorption spectra of (R/S-MBA)$_2$PbI$_4$ films with the introduction of TADDOL molecules. (A, B) Absorption spectra of (R-MBA)$_2$PbI$_4$ films with different concentrations of (A) (+)-TADDOL or (B) (−)-TADDOL. **(C, D)** Absorption spectra of (S-MBA)$_2$PbI$_4$ films with different concentrations of (C) (+)-TADDOL or (D) (−)-TADDOL. Black and red dashed lines show excitonic peak positions before and after adding TADDOLs, respectively, exhibiting hypsochromic shifts.

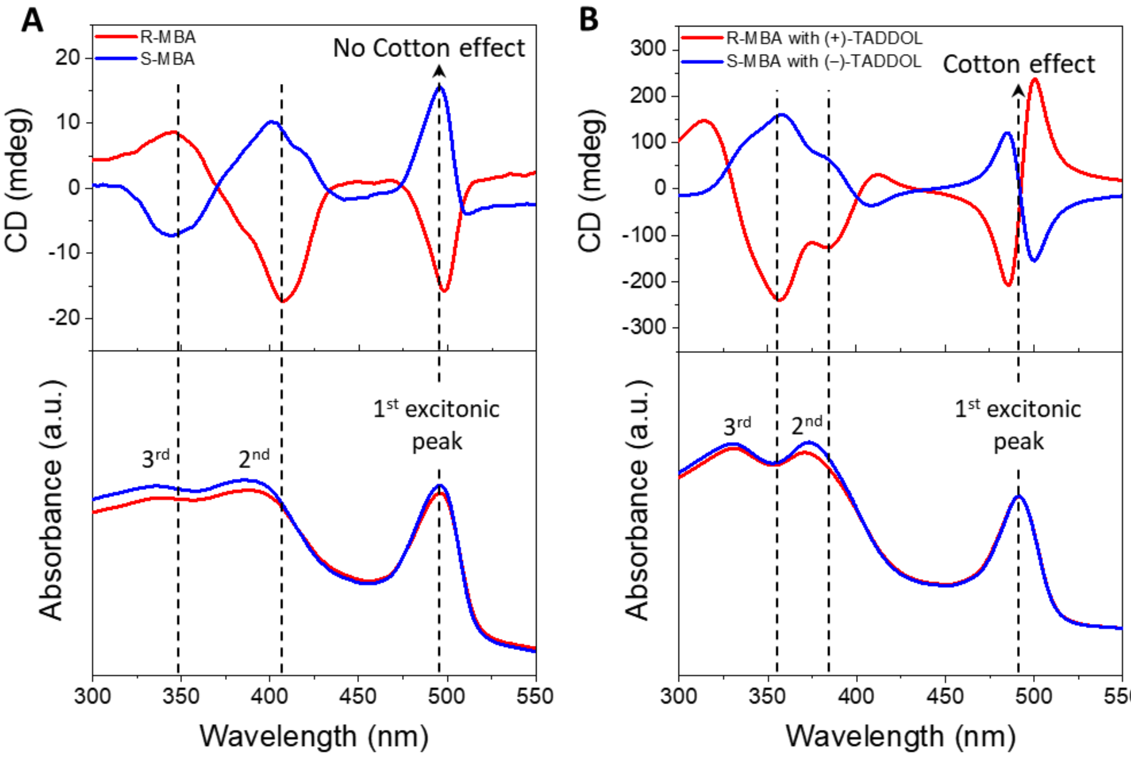

Fig. S13. CD and absorption spectra of chiral 2D OIHP films. (A, B) CD and absorption spectra of chiral 2D OIHPs films (A) w/o TADDOLs or (B) with TADDOLs. Each CD peak can be assigned to corresponding excitonic transition as denoted by vertical dashed lines, implying three excitonic transitions in (*R*/*S*-MBA)$_2$PbI$_4$. From the first excitonic transition, only TADDOL-introduced (*R*-MBA)$_2$PbI$_4$ films exhibited a prominent Cotton effect.

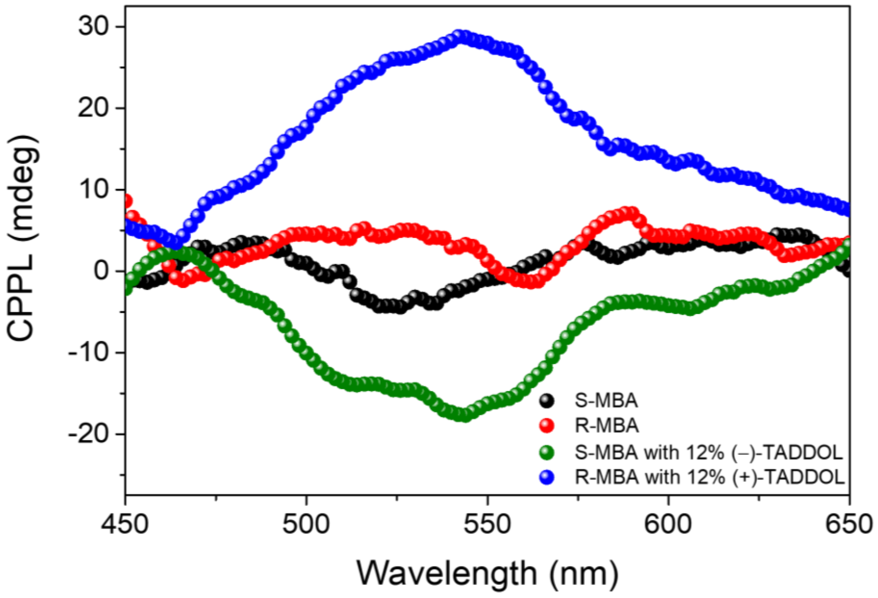

Fig. S14. CPPL spectra from (*R*/*S*-MBA)$_2$PbI$_4$ films with or without TADDOLs.

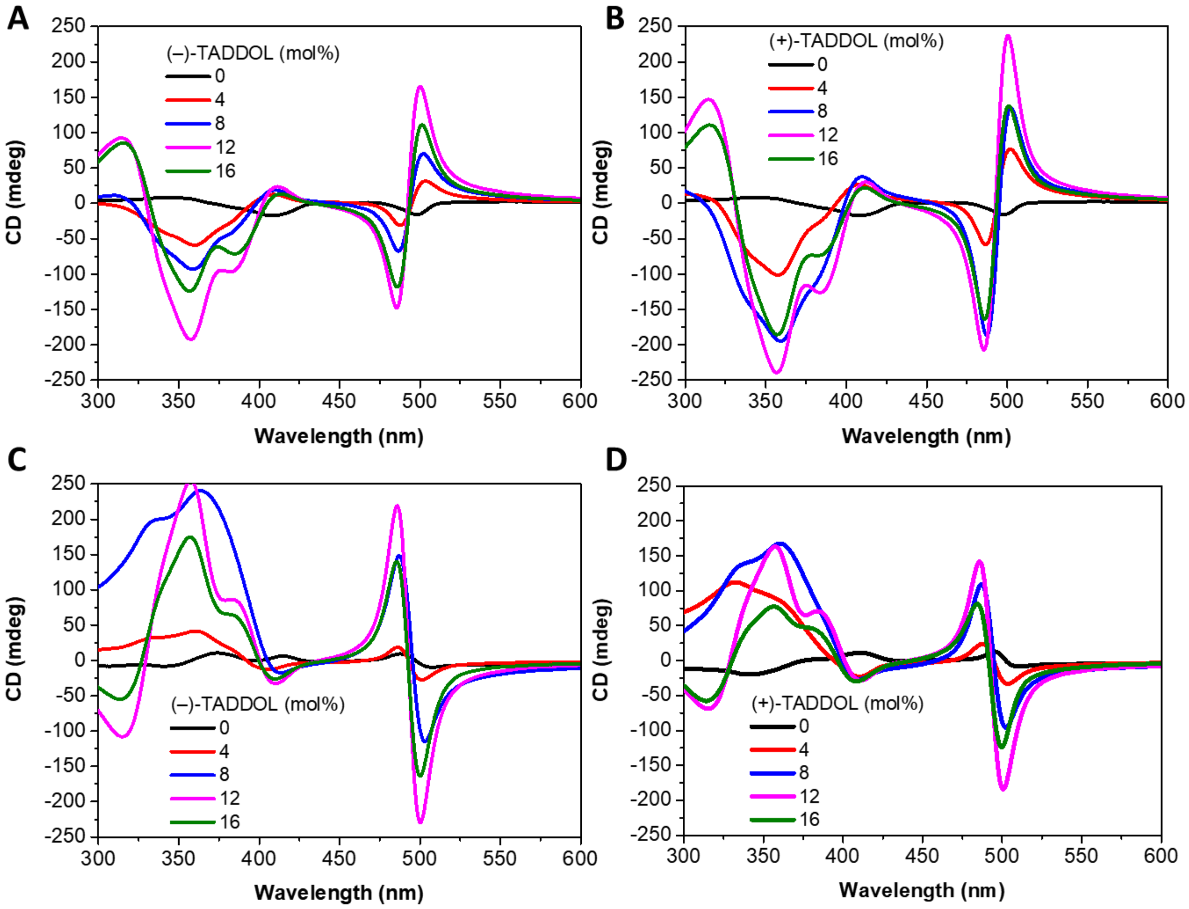

Fig. S15. CD spectra of chiral (*R*/*S*-MBA)$_2$PbI$_4$ with the introduction of TADDOL molecules. (A, B) CD spectra of (*R*-MBA)$_2$PbI$_4$ films with different concentrations of (A) (−)-TADDOL or (B) (+)-TADDOL. **(C, D)** CD spectra of (*S*-MBA)$_2$PbI$_4$ films with different concentrations of (C) (−)-TADDOL or (D) (+)-TADDOL.

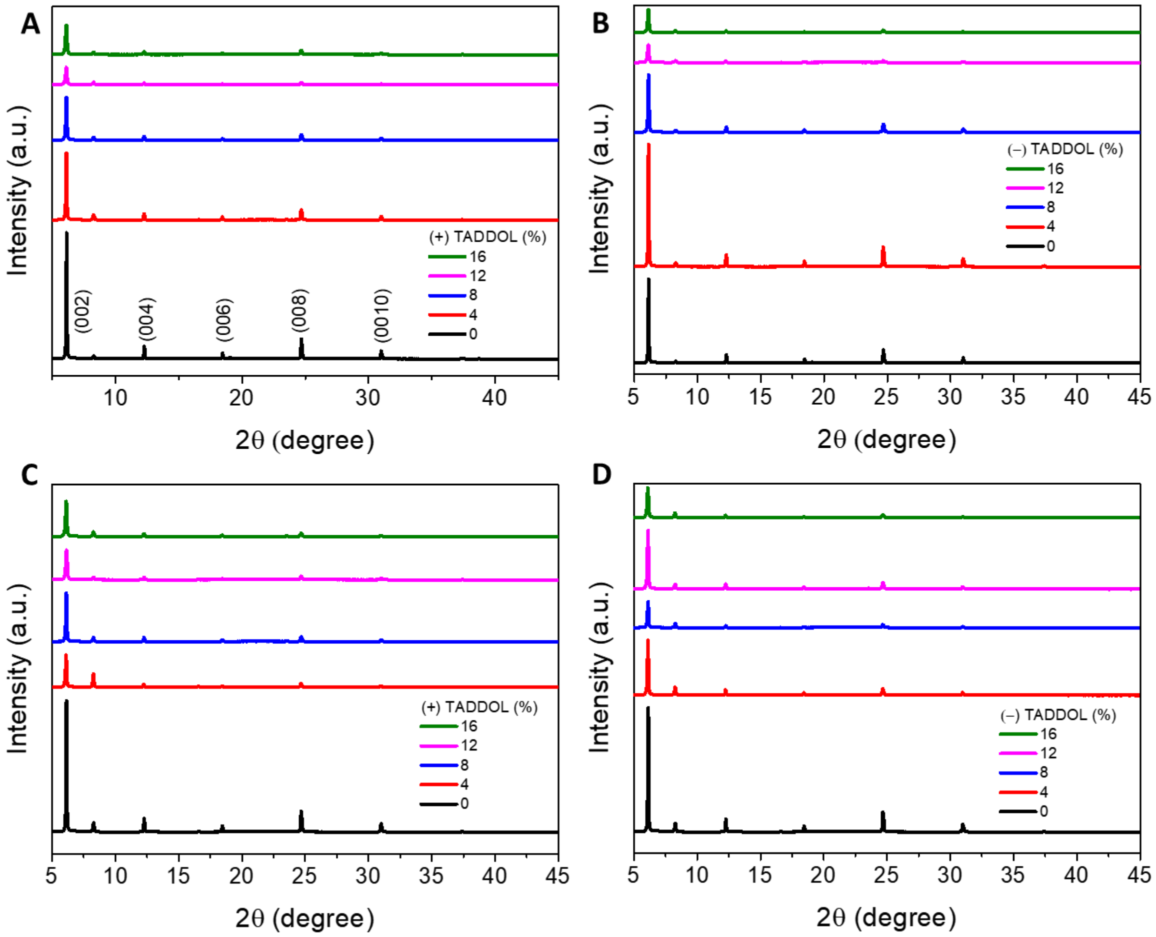

Fig. S16. XRD diffractograms of chiral (*R*/*S*-MBA)$_2$PbI$_4$ with the introduction of TADDOL molecules. (A, B), XRD diffractograms of (*R*-MBA)$_2$PbI$_4$ films with different concentrations of (A) (+)-TADDOL or (B) (−)-TADDOL. **(C, D)**, XRD diffractograms of (*S*-MBA)$_2$PbI$_4$ films with different concentrations of (C) (+)-TADDOL or (D) (−)-TADDOL.

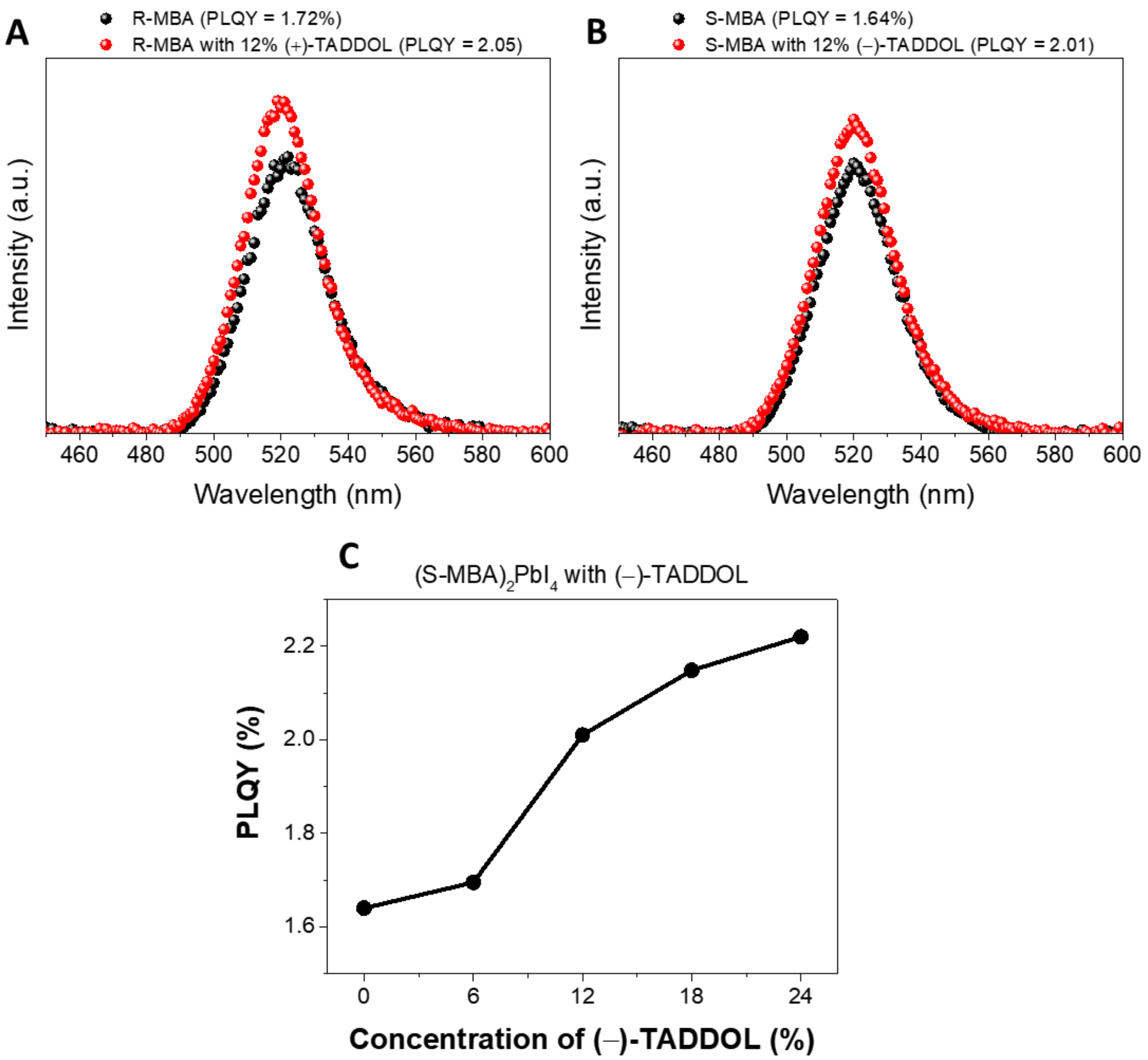

Fig. S17. Steady state PL spectra. PL spectra of **(A)** (R-MBA)$_2$PbI$_4$ and **(B)** (S-MBA)$_2$PbI$_4$ films with or without TADDOLs. Obtained PLQY values are shown at the top. **(C)** PLQY change with different concentrations of TADDOL in (S-MBA)$_2$PbI$_4$, which is consistent with our morphological study.

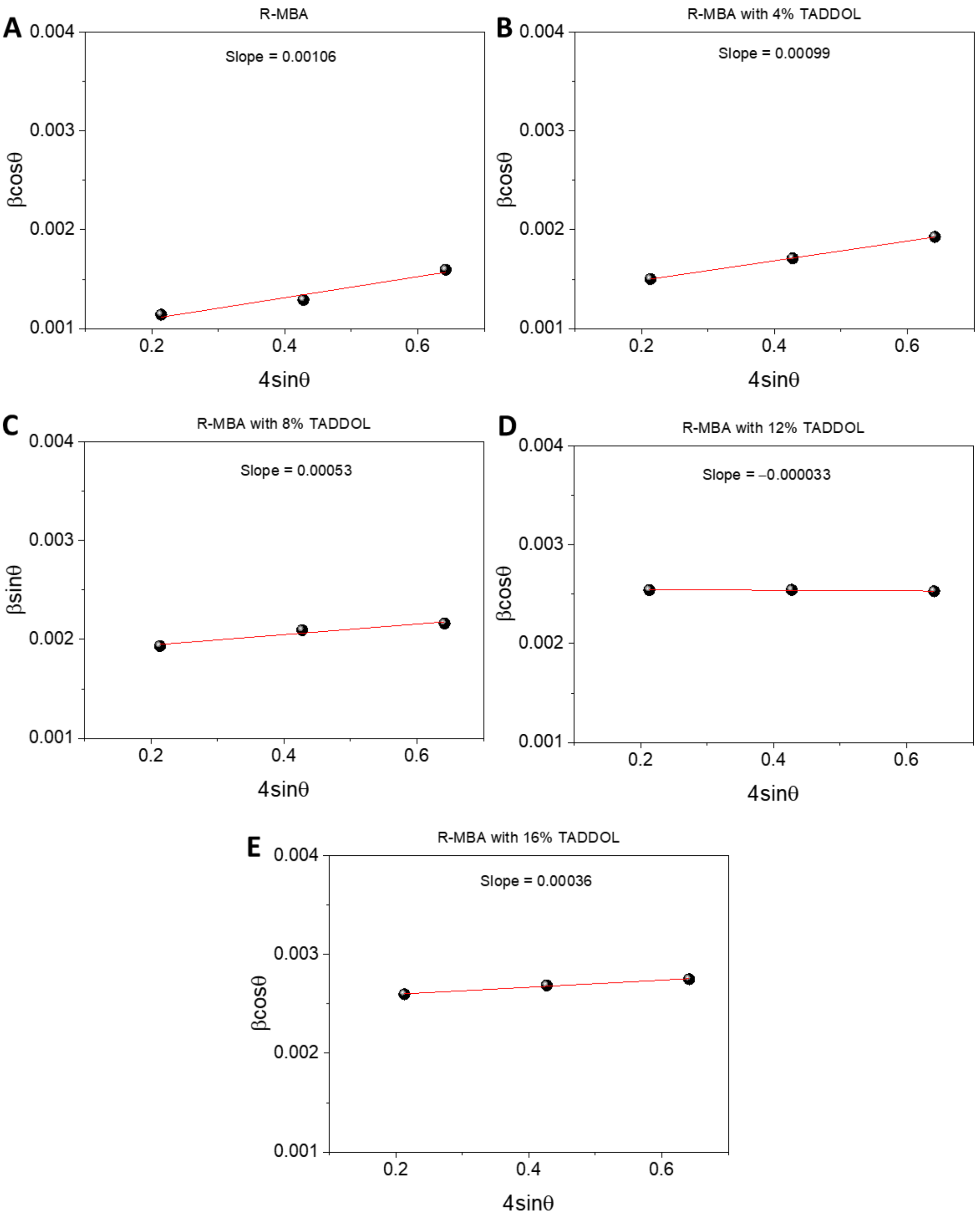

Fig. S18. Williamson-Hall plots of (*R*-MBA)$_2$PbI$_4$ films with different amounts of TADDOLs. (A) 0%, **(B)** 4%, **(C)** 8%, **(D)** 12%, and **(E)** 16%, where the corresponding microstrains were derived from the slopes of Williamson–Hall plots.

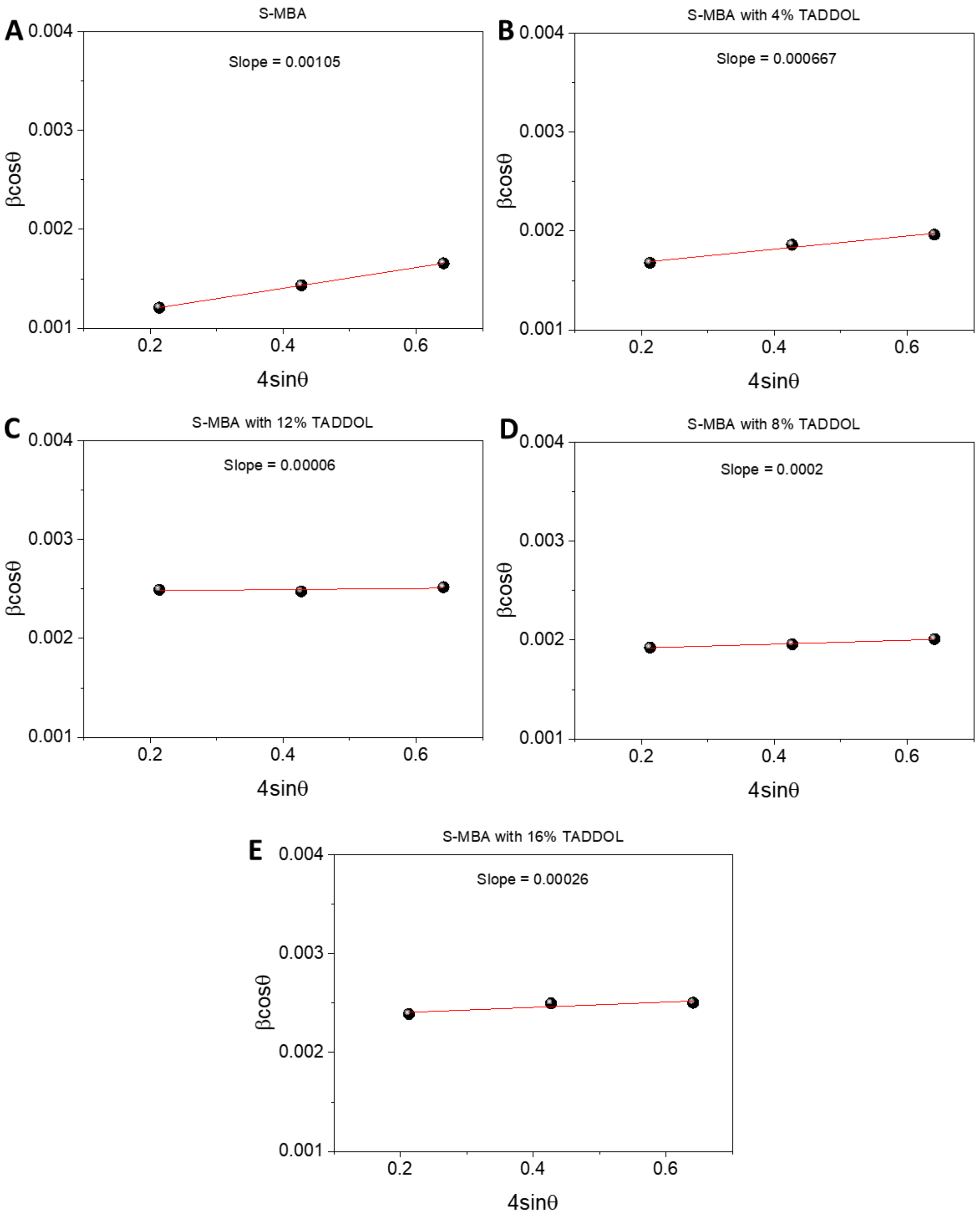

Fig. S19. Williamson-Hall plots of (*S*-MBA)$_2$PbI$_4$ films with different amounts of TADDOLs. (A) 0%, **(B)** 4%, **(C)** 8%, **(D)** 12%, and **(E)** 16%, where the corresponding microstrains were derived from the slopes of Williamson–Hall plots.

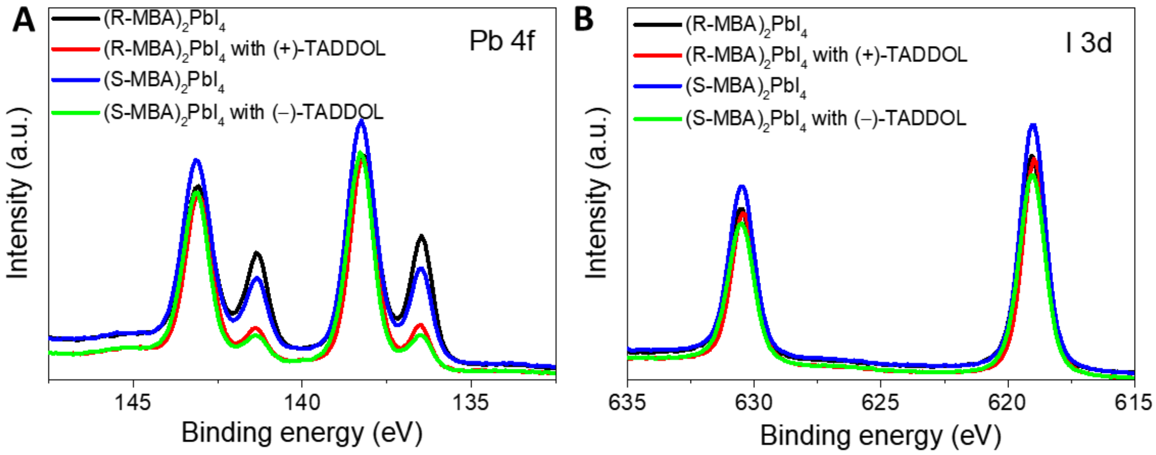

Fig. S20. XPS study on chiral 2D OIHPs films. (A) Pb 4f and **(B)** I 3d XPS spectra for chiral (R/S-MBA)$_2$PbI$_4$ films with or without the introduction of TADDOL molecules. In all XPS spectra with TADDOLs, no noticeable shifts in Pb 4f or I 3d peaks were observed, suggesting insignificant Lewis base–acid interactions between inorganic layers in (R/S-MBA)$_2$PbI$_4$ and TADDOLs.

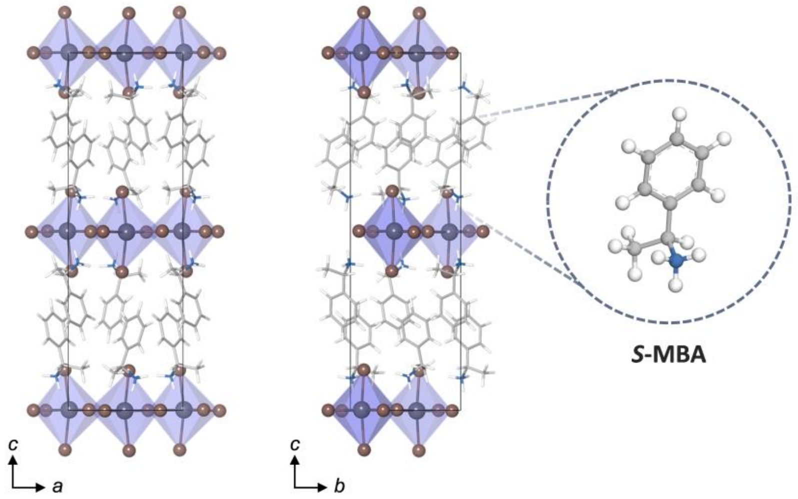

Fig. S21. Optimized bulk structure of (*S*-MBA)$_2$PbI$_4$. Pb, I, C, N, and H are presented by dark gray, brown, gray, blue, and white spheres, respectively. PbI_6 octahedra are shown as translucent purple structures.

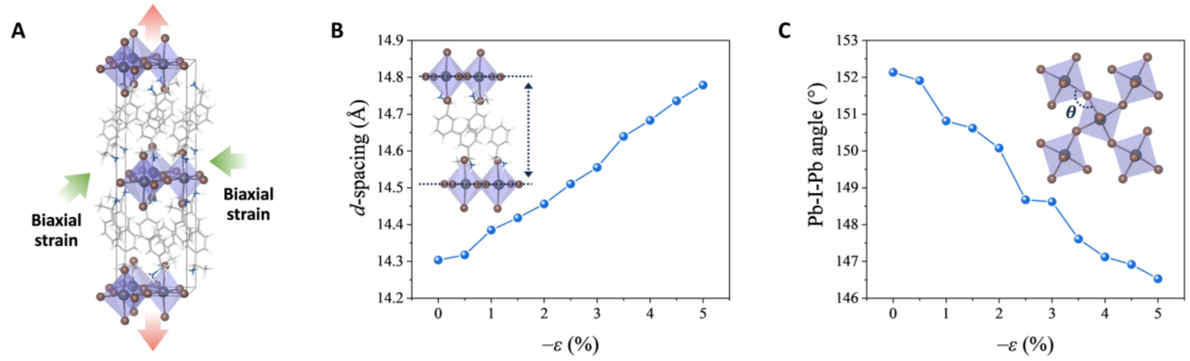

Fig. S22. Bulk structure of (S-MBA)$_2$PbI$_4$ under strain. (A) Biaxial compressive strain applied to the *a*/*b* lattice is indicated by green arrows, while tensile strain induced on the *c* lattice is indicated by red arrows. **(B)** Changes in *d*-spacing according to the applied biaxial strain. **(C)** Changes in average Pb-I-Pb angle according to the applied biaxial strain.

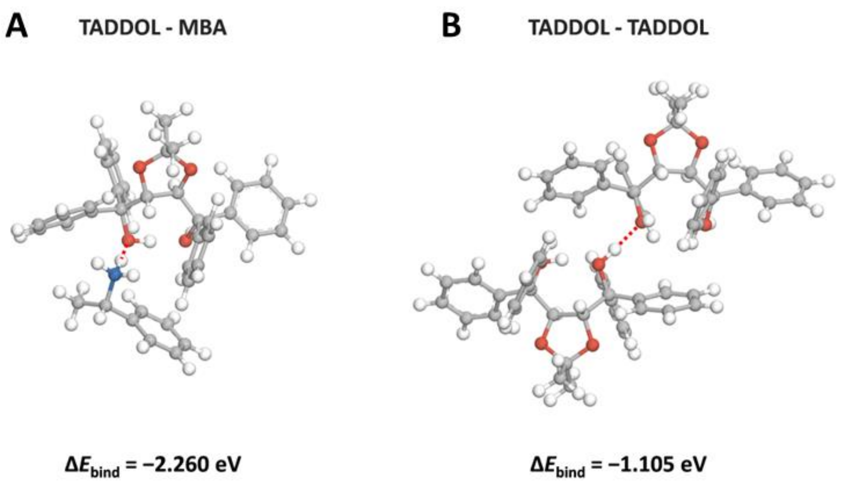

Fig. S23. Binding structure between molecules. (A) Optimized binding structures between the TADDOL and MBA molecules. **(B)** The optimized binding structures among the TADDOL molecules. The favorable binding configuration derived from grand canonical Monte Carlo (GCMC) simulations was utilized. C, O, N, and H are represented by gray, red, blue, and white spheres, respectively. The red dashed line indicates hydrogen bonding.

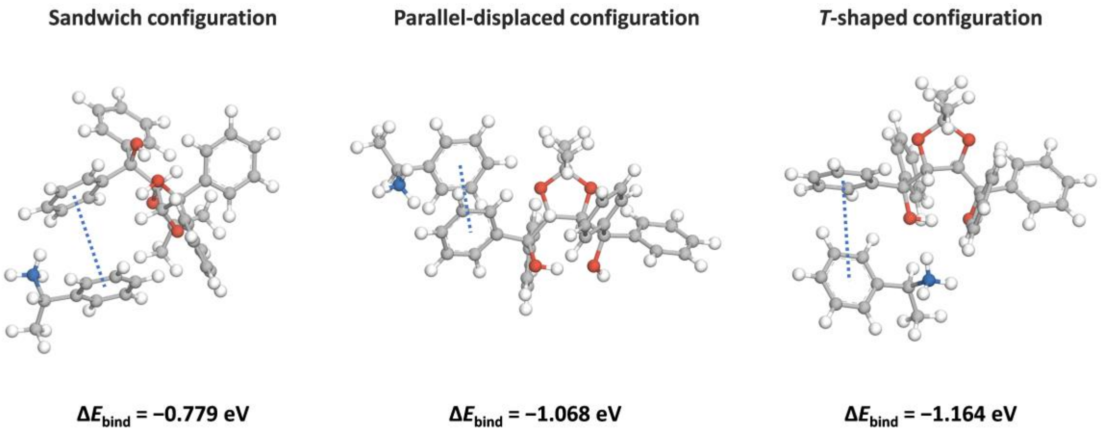

Fig. S24. Binding structure with π–π interaction. Optimized binding structures between TADDOL and MBA molecules according to the three predominant forms of π–π interaction, i.e., sandwich configuration, parallel-displaced configuration, and *T*-shaped configuration (*18*). C, O, N, and H are represented by gray, red, blue, and white spheres, respectively. The blue dotted line indicates the π–π interaction.

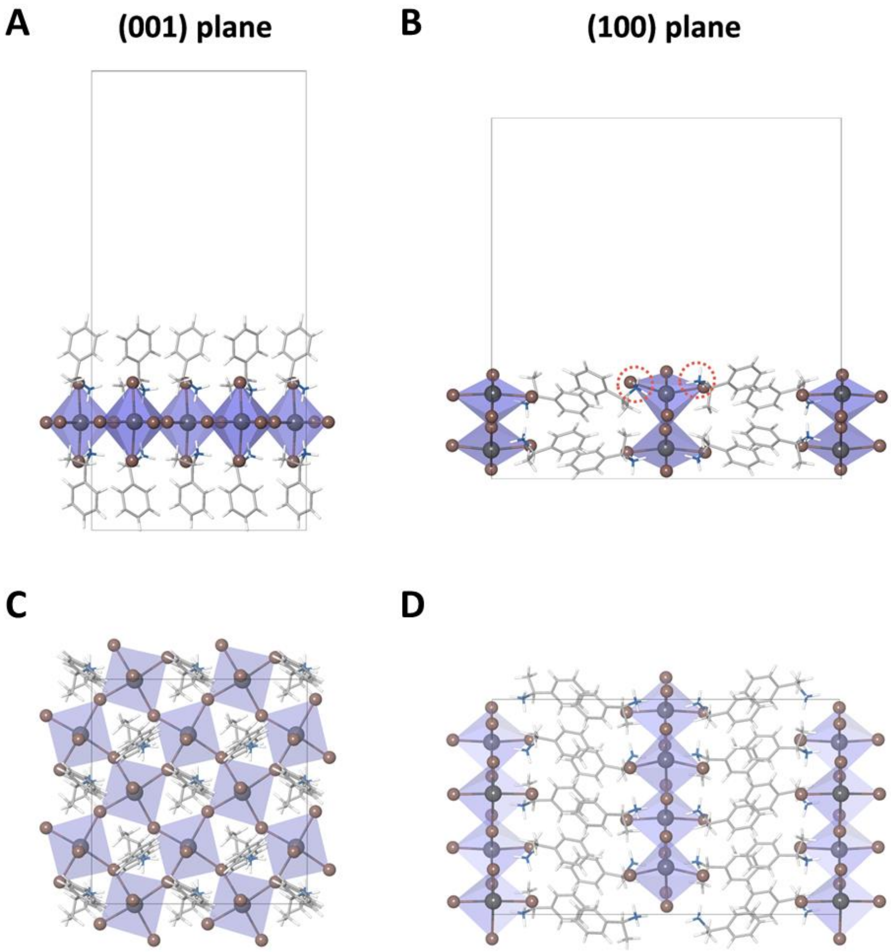

Fig. S25. Optimized surface structures of (*S*-MBA)$_2$PbI$_4$. (A to **D)**, Side views of (A) the optimized (001) and (B) (100) surface structures. (C) Top views of the optimized (001) and (D) (100) surface structures. Pb, I, C, N, and H are represented by dark gray, brown, gray, blue, and white spheres, respectively. The surface-exposed amine groups of MBA are indicated by red dotted circles.

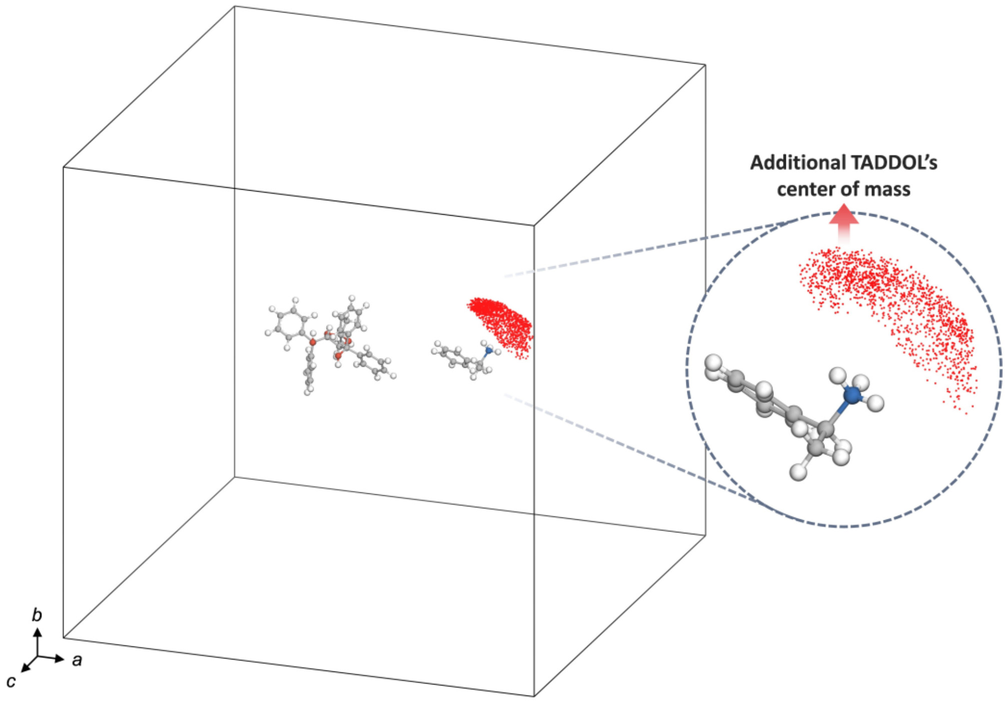

Fig. S26. Probable adsorption sites of extra TADDOL molecule on TADDOL and MBA. The cubic cell was set to 50 × 50 × 50 $Å^3$, composed of TADDOL and MBA molecules, located on the left and right, respectively. The red dots represent the accepted positions of the center of mass of additional TADDOL from GCMC simulations. C, O, N, and H are represented by gray, red, blue, and white spheres, respectively.

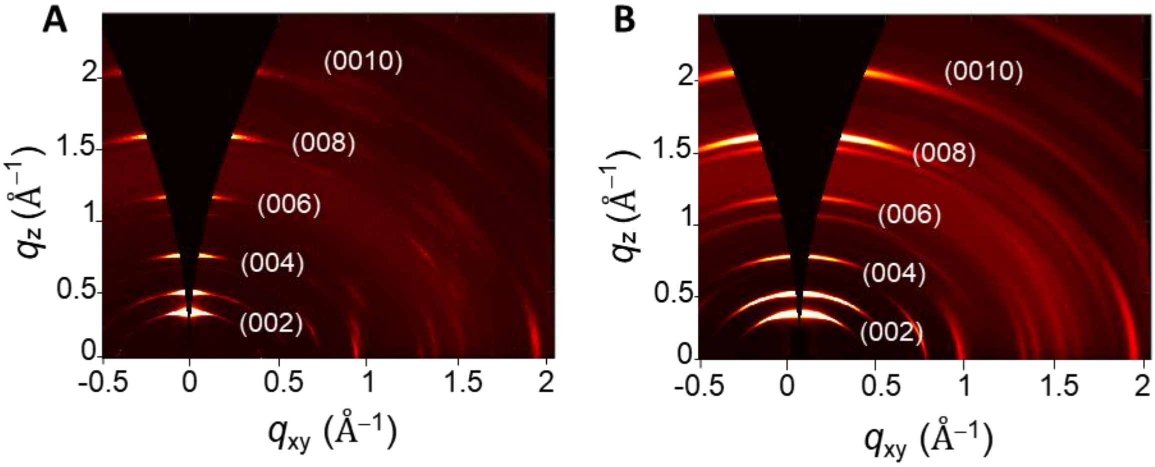

Fig. S27. GIWAXS study on chiral 2D OIHPs. (A, B) GIWAXS patterns of (A) the pristine (*S*-MBA)$_2$PbI$_4$ and (B) TADDOL-introduced (*S*-MBA)$_2$PbI$_4$ films.

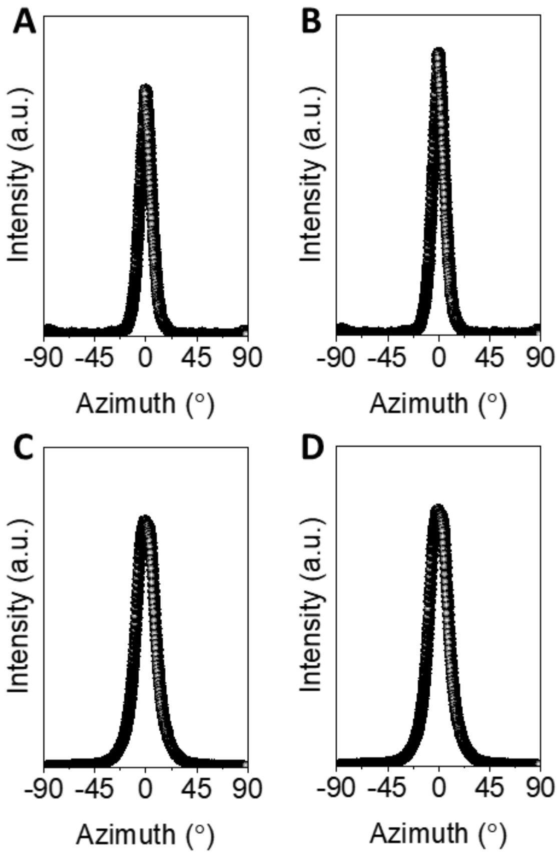

Fig. S28. Crystal orientation of chiral 2D OIHPs. (A to **D),** Integrated intensity plots azimu thally along the ring at q_r assigned to the (002) plane of (A) (*R*-MBA)$_2$PbI$_4$, (B) (*S*-MBA)$_2$PbI$_4$, (C) (*R*-MBA)$_2$PbI$_4$ with TADDOL, and (D) (*S*-MBA)$_2$PbI$_4$ with TADDOL films.

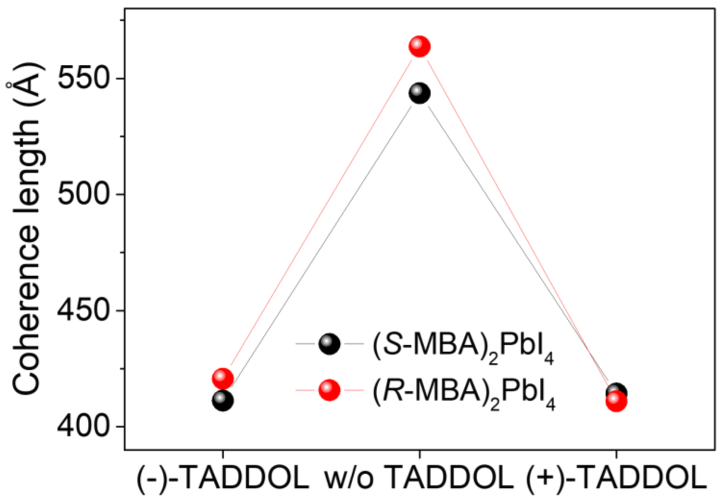

Fig. S29. Coherence lengths of (R/S-MBA)$_2$PbI$_4$ with or without TADDOLs.

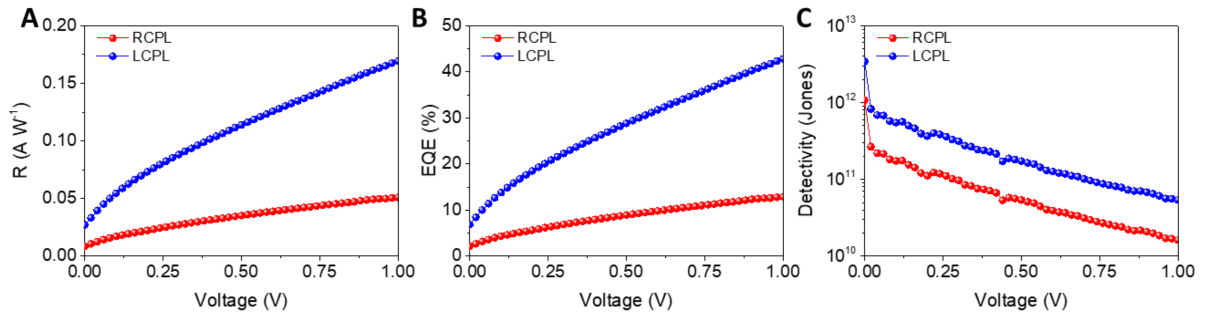

Fig. S30. LCPL and RCPL-dependent photo-responsive characteristics of (−)-TADDOL-introduced (*S*-MBA)$_2$PbI$_4$ detectors. (A) Responsivity, **(B)** EQE, **(C)** specific detectivity (Light intensity of 6 mW cm^{-2}; excitation at a wavelength of 491 nm). The R of the CPL detector was obtained through $R = \frac{I_{\text{light}} - I_{\text{dark}}}{P_{\text{inc}}}$, where I_{light} is the photocurrent produced under illumination, I_{dark} is the dark current, and P_{inc} is the incident light intensity. EQE was defined through $EQE = \frac{(I_{\text{light}} - I_{\text{dark}})hc}{eP_{\text{int}}A\lambda}$, where P_{int} is the incident power density, e is the elementary charge, A is the active area, h is the Planck's constant, c is the speed of light, and λ is the wavelength. Detectivity describes the smallest detectable signal, $D^* = \frac{\sqrt{A}}{NEP}$, where NEP $(\frac{\sqrt{\bar{I}_n^2}}{R\sqrt{\Delta f}})$ is the noise equivalent power, $\bar{I}_n^2$ is the measured noise current, and Δf is the bandwidth. If the major limit to detectivity is shot noise from the current under dark conditions, D^* can be simplified through $D^* = \frac{R}{\sqrt{2e \cdot I_{dark}/A}}$ (*19*).

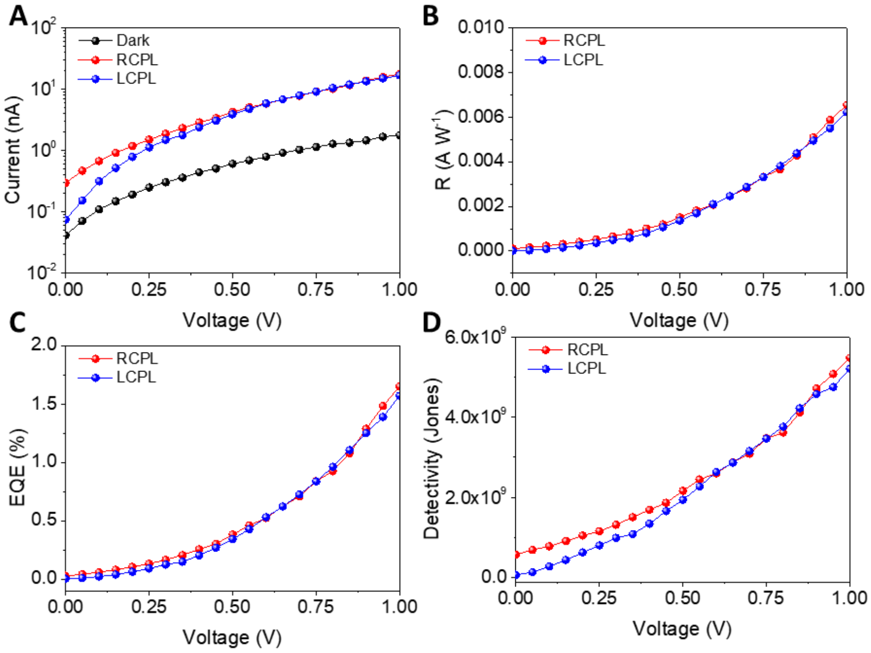

Fig. S31. LCPL and RCPL-dependent photo-characteristics of pristine (*R*-MBA)$_2$PbI$_4$ detectors. (A) *I–V* characteristics under modulation of the SAM of the light. **(B)** Responsivity, **(C)** EQE, **(D)** specific detectivity (Light intensity of 6 mW cm^{-2}; excitation at a wavelength of 491 nm).

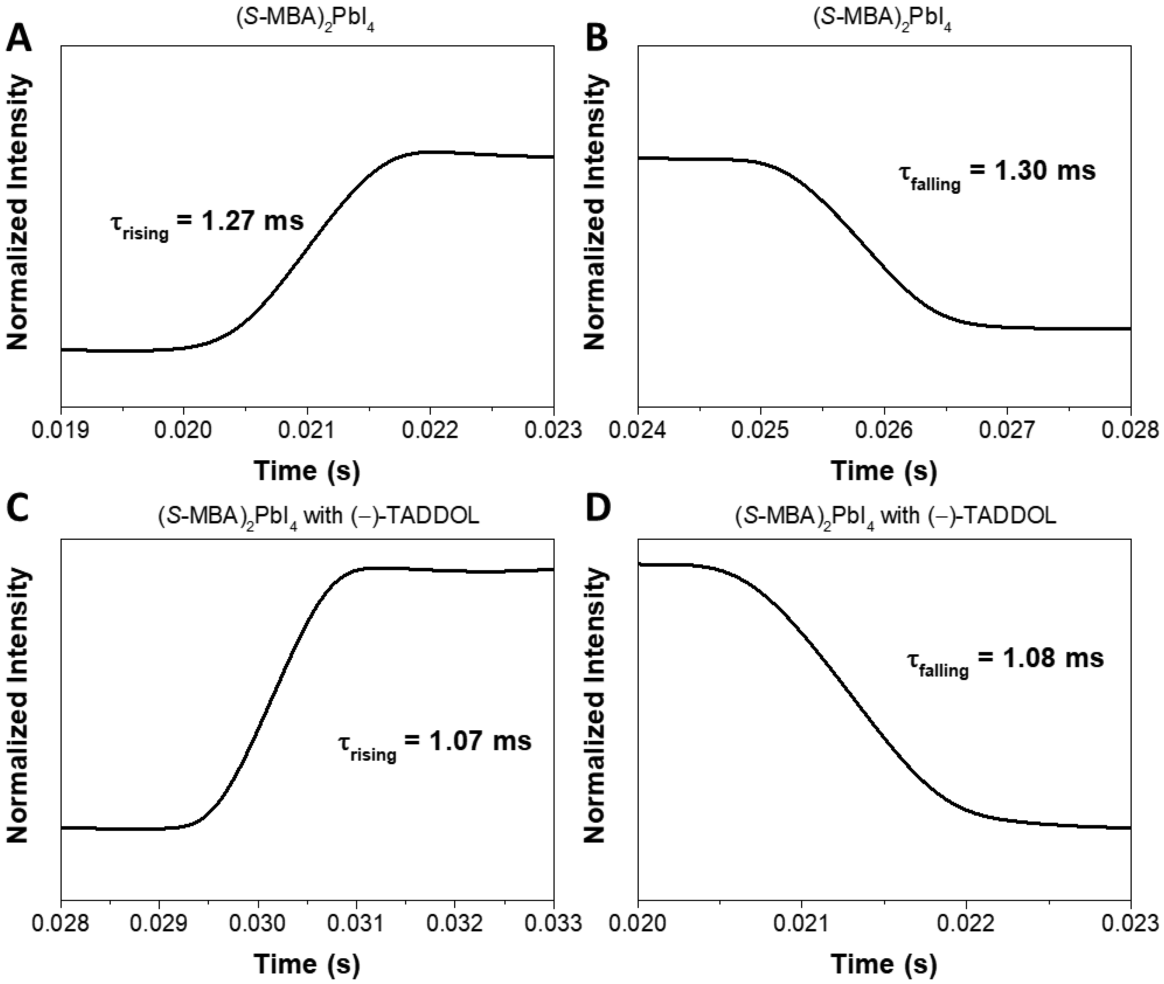

Fig. S32. Rise and fall times of CPL detectors. (A) Rise time of (S-MBA)$_2$PbI$_4$-based CPL detector, **(B)** Fall time of (S-MBA)$_2$PbI$_4$-based CPL detector, **(C)** Rise time of TADDOL-introduced (S-MBA)$_2$PbI$_4$-based CPL detector, **(D)** Fall time of TADDOL-introduced (S-MBA)$_2$PbI$_4$-based CPL detector.

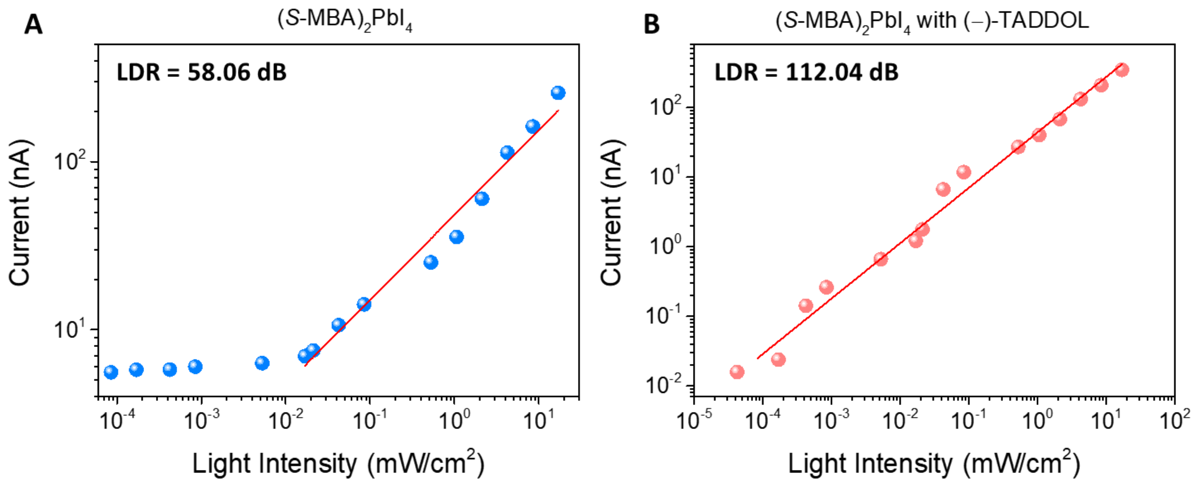

Fig. S33. LDR of CPL detectors. (A) LDR of (*S*-MBA)$_2$PbI$_4$-based CPL detector, **(B)** LDR of TADDOL-introduced (*S*-MBA)$_2$PbI$_4$-based CPL detector.

Table S1. Calculated optoelectronic properties and the transition dipole moments of chiral 2D OIHP films.

	k_{rad} (s)	PLQY (%)	μ (Debye)	g_{PL}	$\|m\|$ (μ_B)	R_{eg} (10^{-40} erg esu cm G^{-1})
R-MBA	2.96×10^7	1.72	1.106 D	0.000531	0.0158	1.624
R-MBA with TADDOL	3.08×10^7	2.05	1.128 D	0.004564	0.1388	12.731
S-MBA	2.92×10^7	1.64	1.100 D	0.000441	0.0131	1.333
S-MBA with TADDOL	3.40×10^7	2.01	1.185 D	0.003507	0.1120	12.311

Table S2. Summarized chiral amplification factors of chiral 2D OIHPs in previous studies.

Perovskite	Methods	Initial $\|g_{CD}\|$	Final $\|g_{CD}\|$	Chiral amplification factor	Inversion of CD peak	Shift of CD peak	Ref.
R/*S*-$MBA_2PbI_{4(0.7)}Br_{4(0.3)}$	Incorporation of Lewis Base Molecule	5.7×10^{-4}	1.0×10^{-3}	1.8-fold	X	X	*20*
R-$MBA_2PbI_{4(1-x)}Br_{4x}$, x=0.325	Confinement of perovskite	3.8×10^{-4}	2.0×10^{-3}	5.3-fold	O	X	*8*
S-$MePEA_2PbBr_4$	Incorporation of achiral organic cation	1.3×10^{-4}	2.7×10^{-4}	2.1-fold	O	X	*21*
(*S*-BrMBA)$_2PbI_4$	Chiral cation doping	1.79×10^{-4}	1.88×10^{-4}	1.1-fold	X	O	*22*
(*R*/*S*-MBA)$_2PbI_4$	Solvent modulation	5.47×10^{-4}	1.39×10^{-3}	2.5-fold	O	O	*23*
(*R*/*S*-MBA)$_2PbI_{4(1-x)}Br_{4x}$	Halide mixing	4.0×10^{-4}	1.0×10^{-3}	2.5-fold	X	O	*24*
(*R*/*S*-PPA)$EAPbBr_4$	Alloying the short chain spacer	1.6×10^{-3}	2.5×10^{-3}	1.6-fold	O	O	*25*
(*R*/*S*-X-MBA)$_2PbI_4$ (X= H, F, Cl, Br, I)	Design of chiral cation	3.0×10^{-4}	3.1×10^{-3}	10.3-fold	O	O	*26*
(*R*/*S*-MBA)$_2PbI_4$	Tuning phase purity	2.5×10^{-4}	2.5×10^{-3}	10-fold	X	X	*27*
(*S*-MBA)$_2PbI_4$	(*S*-MBA)PbI_3 : (*S*-MBA)$_2PbI_4$ mixing	1.0×10^{-3}	3.0×10^{-3}	3-fold (without use of cavity)	X	X	*28*
**(*R*/*S*-MBA)$_2PbI_4$**	**Chiral dopant**	**3.7×10^{-4} (from the average g_{CD} of (*S*-MBA)$_2PbI_4$)**	**5.7×10^{-3} (from the average g_{CD} of (*S*-MBA)$_2PbI_4$ with a chiral dopant)**	**15-fold**	**X**	**X**	**This work**

Table S3. Summarized g_{Ph} of chiral OIHP-based CPL detectors in previous studies.

Perovskite	Dimensionality	g_{Ph}	Driven voltage (V)	Detecting wavelength (nm)	Sensing mode	Ref.
$Cs_{0.05}FA_{0.5}MA_{0.45}Pb_{0.5}Sn_{0.5}I_3$	3D	0.55	0	808	Real time	*29*
$MAPbCl_{0.5}Br_{2.5}$	3D	0.39	5	405	Real time	*30*
(*R*/*S*-BPEA)$EA_6Pb_4Cl_{15}$	3D perovskitoid	0.28	10	320	Non-real time	*31*
(*R*/*S*-NEA)PbI_3	1D	1.85	0.5	395	Non-real time	*32*
(*R*/*S*-α-PEA)PbI_3	1D	0.1	20	395	Non-real time	*33*
(*R*/*S*-NEA)PbI_3	1D	0.294	4	405	Non-real time	*34*
(*R*/*S*-$C_5H_{14}N$)PbI_3	1D	0.23	5	405	Non-real time	*35*
$MBA_4Bi_2Br_{10}$	0D	0.6	0	380	Non-real time	*36*
$CsPbBr_3$+MBA	0D	0.17	1	405	Non-real time	*37*
(*R*/*S*-MBA)$_2CuCl_4$	0D	0.34	0	405	Non-real time	*38*
(*S*/*R*-1-2-NEA)$MAPb_2I_7$	quasi-2D	0.15	40	405	Non-real time	*39*
[(*R*)-MPA]$_2MAPb_2I_7$/$MAPbI_3$	quasi-2D	0.67	0	520	Non-real time	*40*
[(*R*)-*β*-MPA]$_2MAPb_2I_7$	quasi-2D	0.2	10	532	Non-real time	*41*
(*R*/*S*-*β*-MPA)$EA_2Pb_2Br_7$	quasi-2D	0.03	10	800	Non-real time	*42*
(*R*-MPA)$_2MAPb_2I_7$/n-type Si	quasi-2D	0.34	0	520	Non-real time	*43*
(*R*-BPEA)$_2PbI_4$	2D	0.25	10	520	Non-real time	*44*
(*R*/*S*-3AMP)$PbBr_4$	2D	0.22	0	430	Non-real time	*45*
(*R*/*S*-PPA)$EAPbCl_4$	2D	0.4	0	266	Non-real time	*46*
(*R*/*S*-PPA)(PA)$PbBr_4$	2D	0.1	10	405	Non-real time	*47*

(*R*/*S*-MBA)$_2$Pb$_{0.9}$Sn$_{0.1}$I$_4$	2D	0.44	0	500	Non-real time	*48*
(*R*/*S*-*β*-MPA)EAPbBr$_4$	2D	0.19	0	405	Non-real time	*49*
Co^{2+} doped-(PEA)$_2$PbI$_4$	2D	0.41	1	450	Non-real time	*50*
[(*R*/*S*)-*β*-MPA]EAPbBr$_4$	2D	0.19	0	405	Non-real time	*51*
(*R*/*S*-PPA)EAPbBr$_4$	2D	0.42	0	266	Non-real time	*25*
(*R*/*S*-MBA)$_2$PbI$_{4(0.7)}$Br$_{4(0.3)}$	2D	0.27	0	470	Non-real time	*20*
(*R*/*S*-MBA)$_2$PbI$_4$	2D	0.15	5	505	Non-real time	*52*
(*R*/*S*-MBA)$_2$PbI$_4$	2D	0.1	0	486	Non-real time	*53*
(*R*/*S*-MBA)$_2$PbI$_4$	2D	0.24	5	510	Non-real time	*54*
(*R*-BPEA)$_2$PbI$_4$	2D	0.1	10	800	Non-real time	*55*
[(*R*/*S*)-*β*-MPA]$_4$AgBiI$_8$	2D	0.3	0	520	Non-real time	*56*
(*R*/*S*-MBA)$_2$PbI$_4$	2D	0.23	3	520	Non-real time	*57*
**(*R*/*S*-MBA)$_2$PbI$_4$**	2D	**1.16**	**0**	**491**	**Real time**	**This work**

REFERENCES AND NOTES

1. J. Crassous, M. J. Fuchter, D. E. Freedman, N. A. Kotov, J. Moon, M. C. Beard, S. Feldmann, Materials for chiral light control. *Nat. Rev. Mater.* **8,** 365–371 (2023).

2. G. Long, R. Sabatini, M. I. Saidaminov, G. Lakhwani, A. Rasmita, X. Liu, E. H. Sargent, W. Gao, Chiral-perovskite optoelectronics. *Nat. Rev. Mater.* **5**, 423–439 (2020).

3. M. Hu, Y.-X. Yuan, W. Wang, D.-M. Li, H.-C. Zhang, B.-X. Wu, M. Liu, Y.-S. Zheng, Chiral recognition and enantiomer excess determination based on emission wavelength change of AIEgen rotor. *Nat. Commun.* **11**, 161 (2020).

4. L. Pu, Fluorescence of organic molecules in chiral recognition. *Chem. Rev.* **104**, 1687–1716 (2004).

5. I. Song, J. Ahn, H. Ahn, S. H. Lee, J. Mei, N. A. Kotov, J. H. Oh, Helical polymers for dissymmetric circularly polarized light imaging. *Nature* **617**, 92–99 (2023).

6. P. Stachelek, L. MacKenzie, D. Parker, R. Pal, Circularly polarised luminescence laser scanning confocal microscopy to study live cell chiral molecular interactions. *Nat. Commun.* **13**, 553 (2022).

7. D. de Bernardis, F. Piccioli, P. Rabl, I. Carusotto, Chiral quantum optics in the bulk of photonic quantum hall systems. *PRX Quantum* **4**, 030306 (2023).

8. X. Ma, M. Pu, X. Li, C. Huang, Y. Wang, W. Pan, B. Zhao, J. Cui, C. Wang, Z. Zhao, X. Luo, A planar chiral meta-surface for optical vortex generation and focusing. *Sci. Rep.* **5**, 10365 (2015).

9. J. Ma, H. Wang, D. Li, Recent progress of chiral perovskites: Materials, synthesis, and properties. *Adv. Mater.* **33**, e2008785 (2021).

10. A. Pietropaolo, A. Mattoni, G. Pica, M. Fortino, G. Schifino, G. Grancini, Rationalizing the design and implementation of chiral hybrid perovskites. *Chem* **8**, 1231–1253 (2022).

11. J. Ma, C. Fang, C. Chen, L. Jin, J. Wang, S. Wang, J. Tang, D. Li, Chiral 2D perovskites with a high degree of circularly polarized photoluminescence. *ACS Nano* **13**, 3659–3665 (2019).

12. H. Lu, J. Wang, C. Xiao, X. Pan, X. Chen, R. Brunecky, J. J. Berry, K. Zhu, M. C. Beard, Z. V. Vardeny, Spin-dependent charge transport through 2D chiral hybrid lead-iodide perovskites. *Sci. Adv.* **5**, eaay0571 (2019).

13. G. Long, C. Jiang, R. Sabatini, Z. Yang, M. Wei, L. N. Quan, Q. Liang, A. Rasmita, M. Askerka, G. Walters, X. Gong, J. Xing, X. Wen, R. Quintero-Bermudez, H. Yuan, G. Xing, X. R. Wang, D. Song, O. Voznyy, M. Zhang, S. Hoogland, W. Gao, Q. Xiong, E. H. Sargent, Spin control in reduced-dimensional chiral perovskites. *Nat. Photonics* **12**, 528–533 (2018).

14. J. Ahn, E. Lee, J. Tan, W. Yang, B. Kim, J. Moon, A new class of chiral semiconductors: Chiral-organic-molecule-incorporating organic–inorganic hybrid perovskites. *Mater. Horiz.* **4**, 851–856 (2017).

15. Y. Zhao, Y. Qiu, J. Feng, J. Zhao, G. Chen, H. Gao, Y. Zhao, L. Jiang, Y. Wu, Chiral 2D-perovskite nanowires for stokes photodetectors. *J. Am. Chem. Soc.* **143**, 8437–8445 (2021).

16. C. Chen, L. Gao, W. Gao, C. Ge, X. Du, Z. Li, Y. Yang, G. Niu, J. Tang, Circularly polarized light detection using chiral hybrid perovskite. *Nat. Commun.* **10**, 1927 (2019).

17. D. Li, X. Liu, W. Wu, Y. Peng, S. Zhao, L. Li, M. Hong, J. Luo, Chiral lead-free hybrid perovskites for self-powered circularly polarized light detection. *Angew. Chem. Int. Ed.* **60**, 8415–8418 (2021).

18. L. Wang, Y. Xue, M. Cui, Y. Huang, H. Xu, C. Qin, J. Yang, H. Dai, M. Yuan, A chiral reduced-dimension perovskite for an efficient flexible circularly polarized light photodetector. *Angew. Chem. Int. Ed.* **132**, 6504–6512 (2020).

19. A. Ishii, T. Miyasaka, Direct detection of circular polarized light in helical 1D perovskite-based photodiode. *Sci. Adv.* **6**, eabd3274 (2020).

20. J. Son, S. Ma, Y.-K. Jung, J. Tan, G. Jang, H. Lee, C. U. Lee, J. Lee, S. Moon, W. Jeong, A. Walsh, J. Moon, Unraveling chirality transfer mechanism by structural isomer-derived hydrogen bonding interaction in 2D chiral perovskite. *Nat. Commun.* **14**, 3124 (2023).

21. J.-T. Lin, D.-G. Chen, L.-S. Yang, T.-C. Lin, Y.-H. Liu, Y.-C. Chao, P.-T. Chou, C.-W. Chiu, Tuning the circular dichroism and circular polarized luminescence intensities of chiral 2D hybrid organic–

inorganic perovskites through halogenation of the organic ions. *Angew. Chem. Int. Ed.* **60**, 21434–21440 (2021).

22. Y. Peng, X. Liu, L. Li, Y. Yao, H. Ye, X. Shang, X. Chen, J. Luo, Realization of vis–NIR dual-modal circularly polarized light detection in chiral perovskite bulk crystals. *J. Am. Chem. Soc.* **143**, 14077–14082 (2021).

23. J. Zhao, H. Huo, Y. Zhao, Y. Guo, M. Dong, Y. Fu, J. Zhang, Z. Gao, L. Kang, Chiral hybrid perovskites (R-/S-CLPEA)$_4$Bi$_2$I$_{10}$ with enhanced chirality and spin–orbit coupling splitting for strong nonlinear optical circular dichroism and spin selectivity effects. *Chem. Mater.* **35**, 4347–4354 (2023).

24. S. You, P. Yu, T. Zhu, Q. Guan, J. Wu, H. Dai, H. Zhong, Z.-K. Zhu, J. Luo, Alternating chiral and achiral spacers for constructing two-dimensional chiral hybrid perovskites toward circular-polarization-sensitive photodetection. *Mater. Horiz.* **10**, 5307–5312 (2023).

25. L. Yan, M. K. Jana, P. C. Sercel, D. B. Mitzi, W. You, Alkyl–aryl cation mixing in chiral 2D perovskites. *J. Am. Chem. Soc.* **143**, 18114–18120 (2021).

26. R. Lu, Z. Wen, M. Zhao, J. Li, L. Zhang, Y. Yang, H. Jin, Y. Chen, S. Wang, S. Pan, Spacer cation alloying enables markedly improved chiroptical properties of two-dimensional chiral hybrid perovskite nanosheets. *Adv. Opt. Mater.* **11**, 2202290 (2023).

27. S. Ma, Y.-K. Jung, J. Ahn, J. Kyhm, J. Tan, H. Lee, G. Jang, C. U. Lee, A. Walsh, J. Moon, Elucidating the origin of chiroptical activity in chiral 2D perovskites through nano-confined growth. *Nat. Commun.* **13**, 3259 (2022).

28. S. Kim, S.-C. An, Y. Kim, Y. S. Shin, A. A. Antonov, I. C. Seo, B. H. Woo, Y. Lim, M. V. Gorkunov, Y. S. Kivshar, J. Y. Kim, Y. C. Jun, Chiral electroluminescence from thin-film perovskite metacavities. *Sci. Adv.* **9**, eadh0414 (2023).

29. Z. Wang, C.-C. Lin, K. Murata, A. S. A. Kamal, B.-W. Lin, M.-H. Chen, S. Tang, Y.-L. Ho, C.-C. Chen, C.-W. Chen, H. Daiguji, K. Ishii, J.-J. Delaunay, Chiroptical response inversion and enhancement of room-temperature exciton-polaritons using 2D chirality in perovskites. *Adv. Mater.* **35**, e2303203 (2023).

30. T. Zhu, W. Weng, C. Ji, X. Zhang, H. Ye, Y. Yao, X. Li, J. Li, W. Lin, J. Luo, Chain-to-layer dimensionality engineering of chiral hybrid perovskites to realize passive highly circular-polarization-sensitive photodetection. *J. Am. Chem. Soc.* **144**, 18062–18068 (2022).

31. C. U. Lee, S. Ma, J. Ahn, J. Kyhm, J. Tan, H. Lee, G. Jang, Y. S. Park, J. Yun, J. Lee, J. Son, J.-S. Park, J. Moon, Tailoring the time-averaged structure for polarization-sensitive chiral perovskites. *J. Am. Chem. Soc.* **144**, 16020–16033 (2022).

32. J. Ahn, S. Ma, J.-Y. Kim, J. Kyhm, W. Yang, J. A. Lim, N. A. Kotov, J. Moon, Chiral 2D organic inorganic hybrid perovskite with circular dichroism tunable over wide wavelength range. *J. Am. Chem. Soc.* **142**, 4206–4212 (2020).

33. B. Yao, Q. Wei, Y. Yang, W. Zhou, X. Jiang, H. Wang, M. Ma, D. Yu, Y. Yang, Z. Ning, Symmetry-broken 2D lead–tin mixed chiral perovskite for high asymmetry factor circularly polarized light detection. *Nano Lett.* **23**, 1938–1945 (2023).

34. M. K. Jana, R. Song, H. Liu, D. R. Khanal, S. M. Janke, R. Zhao, C. Liu, Z. Valy Vardeny, V. Blum, D. B. Mitzi, Organic-to-inorganic structural chirality transfer in a 2D hybrid perovskite and impact on Rashba-Dresselhaus spin-orbit coupling. *Nat. Commun.* **11**, 4699 (2020).

35. J. Lu, Y. Xue, K. Bernardino, N.-N. Zhang, W. R. Gomes, N. S. Ramesar, S. Liu, Z. Hu, T. Sun, A. F. de Moura, N. A. Kotov, K. Liu, Enhanced optical asymmetry in supramolecular chiroplasmonic assemblies with long-range order. *Science* **371**, 1368–1374 (2021).

36. C.-S. Ho, A. Garcia-Etxarri, Y. Zhao, J. Dionne, Enhancing enantioselective absorption using dielectric nanospheres. *ACS Photonics* **4**, 197–203 (2017).

37. G. Bhat, A. Roth, R. Day, Extrinsic cotton effect and helix-coil transition in a DNA-polycation complex. *Biomolecules* **16**, 1713–1724 (1977).

38. N. Holmgaard List, J. Knoops, J. Rubio-Magnieto, J. Idé, D. Beljonne, P. Norman, M. Surin, M. Linares, Origin of DNA-induced circular dichroism in a minor-groove binder. *J. Am. Chem. Soc.* **139**, 14947–14953 (2017).

39. M. Anyika, H. Gholami, K. D. Ashtekar, R. Acho, B. Borhan, Point-to-axial chirality transfer—A new probe for "sensing" the absolute configurations of monoamines. *J. Am. Chem. Soc.* **136**, 550–553 (2014).

40. G. Williamson, W. Hall, X-ray line broadening from filed aluminium and wolfram. *Acta Metall.* **1**, 22–31 (1953).

41. H. Lu, C. Xiao, R. Song, T. Li, A. E. Maughan, A. Levin, R. Brunecky, J. J. Berry, D. B. Mitzi, V. Blum, M. C. Beard, Highly distorted chiral two-dimensional tin iodide perovskites for spin polarized charge transport. *J. Am. Chem. Soc.* **142**, 13030–13040 (2020).

42. Y.-H. Kim, R. Song, J. Hao, Y. Zhai, L. Yan, T. Moot, A. F. Palmstrom, R. Brunecky, W. You, J. J. Berry, J. L. Blackburn, M. C. Beard, V. Blum, J. M. Luther, The structural origin of chiroptical properties in perovskite nanocrystals with chiral organic ligands. *Adv. Funct. Mater.* **32**, 2200454 (2022).

43. S. Najman, H.-A. Chen, H.-Y. T. Chen, C.-W. Pao, Surface structures and equilibrium shapes of layered 2D Ruddlesden-Popper perovskite crystals from density functional theory calculations. *Mater. Today Commun.* **26**, 101745 (2021).

44. P. Kovaricek, P. Nadazdy, E. Pluharova, A. Brunova, R. Subair, K. Vegso, V. L. P. Guerra, O. Volochanskyi, M. Kalbac, A. Krasnansky, P. Pandit, S. V. Roth, A. Hinderhofer, E. Majkova, M. Jergel, J. Tian, F. Schreiber, P. Siffalovic, Crystallization of 2D hybrid organic–inorganic perovskites templated by conductive substrates. *Adv. Funct. Mater.* **31**, 2009007 (2021).

45. N. S. S. Nizar, M. Sujith, K. Swathi, C. Sissa, A. Painelli, K. G. Thomas, Emergent chiroptical properties in supramolecular and plasmonic assemblies. *Chem. Soc. Rev.* **50**, 11208–11226 (2021).

46. D. di Nuzzo, L. Cui, J. L. Greenfield, B. Zhao, R. H. Friend, S. C. J. Meskers, Circularly polarized photoluminescence from chiral perovskite thin films at room temperature. *ACS Nano* **14**, 7610–7616 (2020).

47. M. S. Alias, I. Dursun, M. I. Saidaminov, E. M. Diallo, P. Mishra, T. K. Ng, O. M. Bakr, B. S. Ooi, Optical constants of $CH_3NH_3PbBr_3$ perovskite thin films measured by spectroscopic ellipsometry. *Opt. Express* **24**, 16586–16594 (2016).

48. G. Kresse, J. Furthmüller, Efficiency of ab-initio total energy calculations for metals and semiconductors using a plane-wave basis set. *Comput. Mater. Sci.* **6**, 15–50 (1996).

49. G. Kresse, J. Furthmüller, Efficient iterative schemes for ab initio total-energy calculations using a plane-wave basis set. *Phys. Rev. B* **54**, 11169–11186 (1996).

50. J. P. Perdew, K. Burke, M. Ernzerhof, Generalized gradient approximation made simple. *Phys. Rev. Lett.* **77**, 3865–3868 (1996).

51. G. Kresse, D. Joubert, From ultrasoft pseudopotentials to the projector augmented-wave method. *Phys. Rev. B* **59**, 1758–1775 (1999).

52. S. Grimme, J. Antony, S. Ehrlich, H. Krieg, A consistent and accurate ab initio parametrization of density functional dispersion correction (DFT-D) for the 94 elements H-Pu. *J. Chem. Phys.* **132**, 154104 (2010).

53. H. J. Monkhorst, J. D. Pack, Special points for Brillouin-zone integrations. *Phys. Rev. B* **13**, 5188–5192 (1976).

54. D. G. Billing, A. Lemmerer, Synthesis and crystal structures of inorganic–organic hybrids incorporating an aromatic amine with a chiral functional group. *CrstEngComm* **8**, 686–695 (2006).

55. D. Systèmes, Biovia materials studio. (Dassault Systèmes, 2019).

56. H. Sun, Z. Jin, C. Yang, R. L. C. Akkermans, S. H. Robertson, N. A. Spenley, S. Miller, S. M. Todd, COMPASS II: Extended coverage for polymer and drug-like molecule databases. *J. Mol. Model.* **22**, 47 (2016).

57. B. Delley, An all-electron numerical method for solving the local density functional for polyatomic molecules. *J. Chem. Phys.* **92**, 508–517 (1990).

58. S. Grimme, Semiempirical GGA-type density functional constructed with a long-range dispersion correction. *J. Comput. Chem.* **27**, 1787–1799 (2006).

59. A. K. Rappe, C. J. Casewit, K. Colwell, W. A. Goddard III, W. M. Skiff, UFF, a full periodic table force field for molecular mechanics and molecular dynamics simulations. *J. Am. Chem. Soc.* **114**, 10024–10035 (1992).

60. R. S. Mulliken, Electronic population analysis on LCAO–MO molecular wave functions. I. *J. Chem. Phys.* **23**, 1833–1840 (1955).

61. Y. H. Lee, I. Song, S. H. Kim, J. H. Park, S. O. Park, J. H. Lee, Y. Won, K. Cho, S. K. Kwak, J. H. Oh, Perovskite granular wire photodetectors with ultrahigh photodetectivity, *Adv. Mater.* **32**, 202002357 (2020).

62. Y. Xie, J. Morgenstein, B. G. Bobay, R. Song, N. A. M. S. Caturello, P. C. Sercel, V. Blum, D. B. Mitzi, Chiral cation doping for modulating structural symmetry of 2D perovskites. *J. Am. Chem. Soc.* **145**, 17831–17844 (2023).

63. H. Zhu, Q. Wang, K. Sun, W. Chen, J. Tang, J. Hao, Z. Wang, J. Sun, W. C. H. Choy, P. Müller-Buschbaum, X. W. Sun, D. Wu, K. Wang, Solvent modulation of chiral perovskite films enables high circularly polarized luminescence performance from chiral perovskite/quantum dot composites. *ACS Appl. Mater. Interfaces* **15**, 9978–9986 (2023).

64. L. Scalon, J. Brunner, M. G. D. Guaita, R. Szostak, M. Albaladejo-Siguan, T. Kodalle, L. A. Guerrero-León, C. M. Sutter-Fella, C. C. Oliveira, Y. Vaynzof, A. F. Nogueira, Tuning phase purity in chiral 2D perovskites. *Adv. Opt. Mater.* **12**, 2300776 (2023).

65. H. Kim, R. M. Kim, S. D. Namgung, N. H. Cho, J. B. Son, K. Bang, M. Choi, S. K. Kim, K. T. Nam, J. W. Lee, J. H. Oh, Ultrasensitive near-infrared circularly polarized light detection using 3D perovskite embedded with chiral plasmonic nanoparticles. *Adv. Sci.* **9**, 2104598 (2022).

66. G. Chen, X. Liu, J. An, S. Wang, X. Zhao, Z. Gu, C. Yuan, X. Xu, J. Bao, H.-S. Hu, J. Li, X. Wang, Nucleation-mediated growth of chiral 3D organic–inorganic perovskite single crystals. *Nat. Chem.* **15**, 1581–1590 (2023).

67. Q. Guan, T. Zhu, Z.-K. Zhu, H. Ye, S. You, P. Xu, J. Wu, X. Niu, C. Zhang, X. Liu, J. Luo, Unprecedented chiral three-dimensional hybrid organic-inorganic perovskitoids. *Angew. Chem. Int. Ed.* **62**, e202307034 (2023).

68. M. Li, F. Fang, X. Huang, G. Liu, Z. Lai, Z. Chen, J. Hong, Y. Chen, R.-J. Wei, G.-H. Ning, K. Leng, Y. Shi, B. Tian, Chiral ligand-induced structural transformation of low-dimensional hybrid perovskite for circularly polarized photodetection. *Chem. Mater.* **34**, 2955–2962 (2022).

69. Y. Zhao, X. Li, J. Feng, J. Zhao, Y. Guo, M. Yuan, G. Chen, H. Gao, L. Jiang, Y. Wu, Chiral 1D perovskite microwire arrays for circularly polarized light detection. *Giant* **9**, 100086 (2022).

70. A. Maiti, A. J. Pal, Spin-selective charge transport in lead-free chiral perovskites: The key towards high-anisotropy in circularly-polarized light detection. *Angew. Chem. Int. Ed.* **61**, e202214161 (2022).

71. J. Hao, H. Lu, L. Mao, X. Chen, M. C. Beard, J. L. Blackburn, Direct detection of circularly polarized light using chiral copper chloride–carbon nanotube heterostructures. *ACS Nano* **15**, 7608–7617 (2021).

72. T. Liu, W. Shi, W. Tang, Z. Liu, B. C. Schroeder, O. Fenwick, M. J. Fuchter, High responsivity circular polarized light detectors based on quasi two-dimensional chiral perovskite films. *ACS Nano* **16**, 2682–2689 (2022).

73. X. Zhang, X. Liu, L. Li, C. Ji, Y. Yao, J. Luo, Great amplification of circular polarization sensitivity via heterostructure engineering of a chiral two-dimensional hybrid perovskite crystal with a three-dimensional $MAPbI_3$ Crystal. *ACS Cent. Sci.* **7**, 1261–1268 (2021).

74. W. Wu, X. Shang, Z. Xu, H. Ye, Y. Yao, X. Chen, M. Hong, J. Luo, L. Li, Toward efficient two-photon circularly polarized light detection through cooperative strategies in chiral quasi-2D perovskites. *Adv. Sci.* **10**, 2206070 (2023).

75. J. Wu, X. Zhang, S. You, Z.-K. Zhu, T. Zhu, Z. Wang, R. Li, Q. Guan, L. Liang, X. Niu, J. Luo, Low detection limit circularly polarized light detection realized by constructing chiral perovskite/Si heterostructures. *Small* **19**, e2302443 (2023).

76. X. Zhang, H. Ye, L. Liang, X. Niu, J. Wu, J. Luo. Direct detection of near-infrared circularly polarized light via precisely designed chiral perovskite heterostructures. *ACS Appl. Mater. Interfaces* **14**, 36781–36788 (2022).

77. C.-C. Fan, X.-B. Han, B.-D. Liang, C. Shi, L.-P. Miao, C.-Y. Chai, C.-D. Liu, Q. Ye, W. Zhang, Chiral rashba ferroelectrics for circularly polarized light detection. *Adv. Mater.* **34**, e2204119 (2022).

78. T. Zhu, H. Wu, C. Ji, X. Zhang, Y. Peng, Y. Yao, H. Ye, W. Weng, W. Lin, J. Luo, Polar photovoltaic effect in chiral alternating cations intercalation-type perovskites driving self-powered ultraviolet circularly polarized light detection. *Adv. Opt. Mater.* **10**, 2200146 (2022).

79. W. Wu, L. Li, D. Li, Y. Yao, Z. Xu, X. Liu, M. Hong, J. Luo, Tailoring the distinctive chiral-polar perovskites with alternating cations in the interlayer space for self-driven circularly polarized light detection. *Adv. Opt. Mater.* **10**, 2102678 (2022).

80. Y. Liu, Y. Jiang, Z. Xu, L. Li, D. Zhang, W. Zheng, D. Liang, B. Zheng, H. Liu, X. Sun, C. Zhu, L. Lin, X. Zhu, H. Duan, Q. Yuan, X. Wang, S. Wang, D. Li, A. Pan, Magnetic doping induced strong circularly polarized light emission and detection in 2D layered halide perovskite. *Adv. Opt. Mater.* **10**, 2200183 (2022).

81. Y. Zhao, M. Dong, J. Feng, J. Zhao, Y. Guo, Y. Fu, H. Gao, J. Yang, L. Jiang, Y. Wu, Lead-free chiral 2D double perovskite microwire arrays for circularly polarized light detection. *Adv. Opt. Mater.* **10**, 2102227 (2022).

82. J. Wang, H. Lu, X. Pan, J. Xu, H. Liu, X. Liu, D. R. Khanal, M. F. Toney, M. C. Beard, Z. V. Vardeny, Spin-dependent photovoltaic and photogalvanic responses of optoelectronic devices based on chiral two-dimensional hybrid organic–inorganic perovskites. *ACS Nano* **15**, 588–595 (2021).

83. Z. Liu, C. Zhang, X. Liu, A. Ren, Z. Zhou, C. Qiao, Y. Guan, Y. Fan, F. Hu, Y. S. Zhao, Chiral hybrid perovskite single-crystal nanowire arrays for high-performance circularly polarized light detection. *Adv. Sci.* **8**, 2102065 (2021).

84. J. Wang, C. Fang, J. Ma, S. Wang, L. Jin, W. Li, D. Li, Aqueous synthesis of low-dimensional lead halide perovskites for room-temperature circularly polarized light emission and detection. *ACS Nano* **13**, 9473–9481 (2019).
